# Revealing two distinct molecular binding modes in polyethyleneimine–DNA polyplexes using infrared spectroscopy†

**DOI:** 10.1039/d5sm00213c

**Published:** 2025-04-26

**Authors:** Rusul Mustafa, Danielle Diorio, Madeline Harper, David Punihaole

**Affiliations:** a Department of Chemistry, University of Vermont 82 University Place Burlington Vermont USA David.Punihaole@uvm.edu

## Abstract

In this study, we use infrared spectroscopy to investigate the molecular binding modes of DNA with linear and branched polyethylenimine (LPEI and BPEI). PEI-based polymers are widely studied as non-viral gene delivery vectors, but their low transfection efficiency limits their clinical success. One key factor affecting their performance is how they bind DNA as it directly impacts the packaging, protection, and release of the cargo in cells. While PEI–DNA binding has traditionally been viewed through the lens of electrostatics, computational models suggest additional binding mechanisms may be involved. Our findings reveal that LPEI and BPEI exhibit two distinct molecular binding modes, which influence DNA packaging into polyplexes. Identifying these binding modes provides critical insights into polymer complexation mechanisms to nucleic acids that can guide the rational design of more efficient and versatile PEI-based gene delivery systems.

## Abstract 2

Proposed binding models for LPEI and BPEI polyplexes to guide the rational design of more effective PEI-based delivery systems.

## Graphical abstract

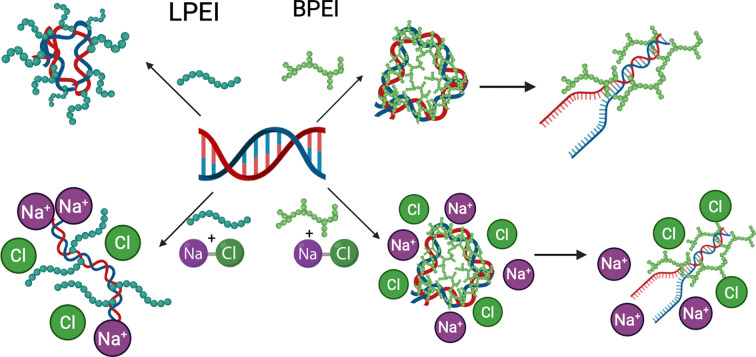

## 1. Introduction

Polymers are promising non-viral delivery vehicles for gene editing and therapy applications, offering a safer and more versatile alternative to viral vectors, which face challenges like immunogenicity, limited capacity, and high production costs.^1,2^ Polymers bind and compact DNA or RNA cargo into nanoparticle complexes, called polyplexes, for delivery into cells.^3^ Among the most popular polymer systems is polyethyleneimine (PEI), sold commercially as jetPEI. This polymer is widely used as a basic scaffold for delivery systems employed in routine transfection and gene editing applications.^4–8^

Despite their potential, PEI-based systems still face significant limitations in achieving the high delivery efficiencies required for clinical use.^9^ A critical factor influencing efficiency is the formation and stability of polyplexes.^10,11^ Strong binding and efficient nucleic acid release must be carefully balanced because overly stable polyplexes can hinder intracellular release, while weak complexes fail to protect and transport cargo effectively into cells.^12–14^ Previous studies have predominantly focused on optimizing transfection efficiency only by adjusting molecular weight,^15,16^ nitrogen-to-phosphate (N/P) ratios,^17^ or complex formation conditions.^18–20^

Other investigations have examined PEI–DNA complexation mechanisms by examining factors such as binding strength, stability, and structure. For example, Ketola *et al.*^21–23^ used fluorescence spectroscopy to reveal distinct differences in DNA cargo binding for linear and branched PEI (LPEI and BPEI, respectively). Similarly, there are some reports that have examined PEI binding mechanisms to DNA by utilizing circular dichroism (CD) and isothermal titration calorimetry (ITC).^24,25^ These studies show that DNA exhibits significant conformational changes upon binding to PEI. Other studies also suggest that BPEI offers better DNA protection than LPEI, but lower delivery efficiency.^26^ However, these studies do not provide a molecular-level picture of the binding modes or conformational dynamics of LPEI and BPEI polyplexes. Developing this molecular-level understanding is crucial for being able to tune the interactions of polymeric carriers. This can design PEI systems to deliver different types of cargo, form more stable polyplexes, offer better cargo protection, or release their cargo more efficiently.

Building on this need for deeper molecular insights, recent work by Reineke and coworkers^14,27^ introduced versatile quinine-based polymers capable of delivering multiple cargos, including plasmid DNA, mRNA, and CRISPR/Cas9 machinery. Their studies suggest that the ability of these delivery systems to robustly accommodate and deliver these different types of cargoes is facilitated by their ability to bind them through multiple mechanisms, including electrostatic, π-stacking, and hydrogen bonding interactions.^14,27,28^ This insight opens new avenues for investigating similar phenomena in PEI-based systems, whose interactions with nucleic acid cargo have historically been assumed to be only electrostatic in nature. Interestingly, several computational studies and experimental reports utilizing ITC have predicted that BPEI might engage in additional DNA binding modes that go beyond electrostatics.^29–31^ However, there has not been any direct experimental structure-based evidence to confirm this, especially on larger PEI delivery systems.

To address this gap, we investigated the binding modes and macromolecular structure of PEI–DNA polyplexes using infrared (IR) spectroscopy and transmission electron microscopy (TEM). IR spectroscopy probes vibrations that report on bond-specific interactions and conformational changes, while TEM provides insights into polyplex morphology. Together, these methods offer a comprehensive molecular-level perspective of PEI–DNA dynamics.

Using TEM, we first examine the morphologies of LPEI and BPEI polyplexes, followed by UV-Vis absorption spectroscopy to analyze the complexation of these polymers to DNA. We then apply Fourier transform IR (FTIR) spectroscopy with multivariate curve resolution (MCR) analysis to differentiate between the binding modes of LPEI and BPEI polyplexes. Our comprehensive approach elucidates new insights into the molecular-level binding mechanisms of PEI systems to DNA, which we anticipate will help guide the development of more efficient and versatile PEI-based gene delivery systems.

## 2. Materials and methods

### 2.1. Materials

DNA from herring sperm (<50 bp), linear PEI hydrochloride (Mn. 15 000 Da), sodium chloride (NaCl, molecular biology grade, ≥99% purity), sodium phosphate monobasic (molecular biology grade, anhydrous, ≥98% purity), sodium phosphate dibasic (molecular biology grade) were all purchased from Sigma Aldrich. Phosphate buffer saline (PBS) tablets were purchased from Fisher Scientific. Branched PEI (Mn. 10 000 Da) was purchased from Polysciences. Buffer solutions were prepared in MilliQ (18.2 MΩ) water. The ratio of the UV absorption at 260 nm (*A*_260nm_) and 280 nm (*A*_280nm_) for the DNA samples were *A*_260nm_/*A*_280nm_ = 1.8, indicative of “pure” DNA.^32^

### 2.2. Polyplex sample preparation

For binding experiments involving UV-Vis absorption spectroscopy, we prepared stock solutions of polymer and DNA in PBS (pH 7.4) with final concentrations of 0.5 μM for DNA, 20 μM for linear PEI, and 50 μM for branched PEI. We then added aliquots of polymer stock solutions with different volumes to DNA stocks to create solutions with N/P ratios (the molar ratios of polymer amines to DNA phosphate) ranging from 0–10. Samples became slightly turbid upon adding polymer stock solutions to DNA stocks, indicating polyplex formation. We maintained the pH of all samples at 7.4. Since the polymer volumes were very small, the final concentration of DNA was maintained around 0.5 μM (see the ESI,† for details). Additionally, we prepared solutions of only polymer, without DNA, with similar final polymer concentrations as the polyplex samples. All samples were then incubated at room temperature for 24 hours to equilibrate prior to performing binding affinity experiments.

For FTIR experiments, we prepared stock solutions of polymer and DNA in 0.01 M phosphate buffer (pH 7.4), with and without the addition of NaCl. For the screening experiment, NaCl was added to the phosphate buffer to achieve final concentrations of 50, 100, 150, 200, and 250 mM. The final pH for the phosphate buffer after the addition of NaCl was 7.4. Using these buffers, we prepared polymer and DNA stock solutions at concentrations of 2 mM (DNA), 3 mM (LPEI), and 5 mM (BPEI). The polymer stocks were further diluted to obtain series of solutions with specific working concentrations. These were added dropwise to DNA solutions while gently mixing, to form polyplex samples with N/P ratios ranging from 0.25 to 3.0. The final DNA concentration in all polyplex samples was adjusted to 1 mM. The final pH of all samples was approximately 5.0 due to the high concentrations of polymer and DNA. DNA-only samples (with and without NaCl) were also prepared at 1 mM as references. Additionally, polymer-only controls were prepared at final concentrations of 1.8 mM for LPEI and 2.6 mM for BPEI. All samples were allowed to equilibrate at room temperature for one hour prior to FTIR measurements.

### 2.3. UV absorption spectroscopy

We obtained UV absorption spectra using an Agilent Cary UV-Vis-NIR Spectrophotometer equipped with a tungsten halogen and deuterium arc lamp. Spectra were collected between 200–350 nm using a scan rate of 600 nm min^−1^.

### 2.4. FTIR spectroscopy measurements

We collected IR absorption spectra using a Bruker INVENIO Fourier transform IR spectrometer with a concentratIR2 multiple reflection silicon ATR head purchased from Harrick Scientific. This ATR unit is designed for micro-liquid samples and has eleven internal reflections with a nominal incident angle of 30°. We performed background measurements first and then added 60 μL of sample on the ATR sampling area. We measured all spectra in the 1100–1800 cm^−1^ range with 4 cm^−1^ resolution and 128 scans in transmission mode. We then converted the spectra to absorption mode using the Bruker software.

### 2.5. Transmission electron microscopy (TEM) imaging

A 5 μL aliquot of sample was placed on freshly glow discharged carbon-coated grid and incubated for 2 min. Following incubation, the grid was then washed 5× with water and wicked dry with filter paper. The samples were then stained using 3% (w/v) uranyl acetate for 2 min. Excess stain was then removed from the sample grid by wicking with filter paper. The grids were air dried in a dust free environment. TEM imaging was then performed using a JEOL 1400 electron microscope operating at 120 kV, equipped with a AMT XR611 CCD camera.

### 2.6. Data processing

We processed all FTIR and UV-Vis spectra using custom MATLAB scripts written in-house (see ESI†). We performed baseline correction and background subtraction for all spectra. To compare the spectra, we additionally normalized FTIR spectra to the total integrated intensity. We used Prism GraphPad software to fit the binding affinity data collected with UV-Vis spectroscopy. Multivariate curve resolution (MCR) analysis was performed using the MCR-ALS software developed by Felten *et al.*^33^

## 3. Results and discussion

### 3.1. Polymer systems

**Fig. 1:**
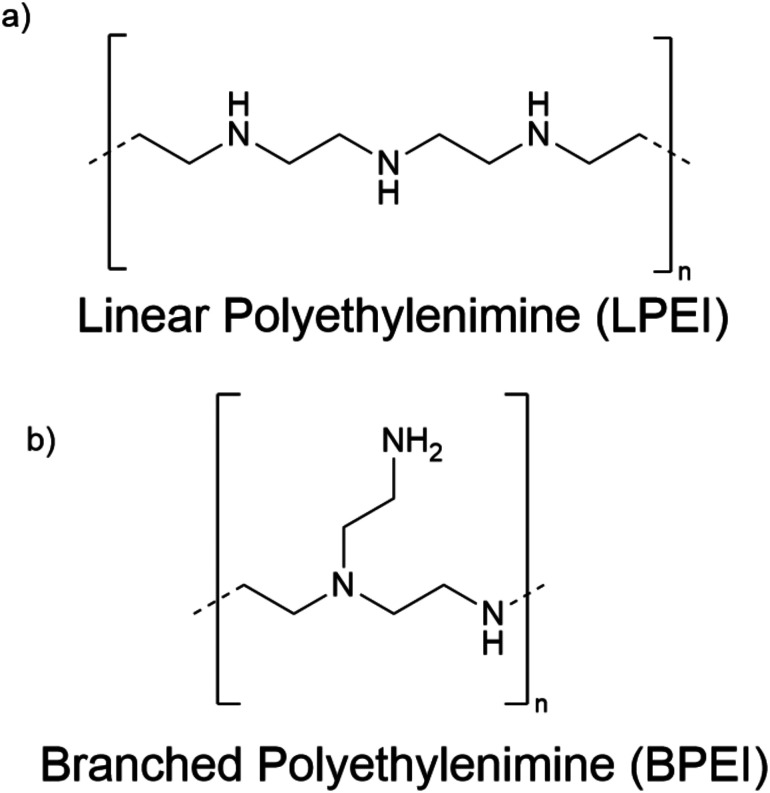
Panels (a) and (b) represent the chemical structures of the polymer systems used in this study.

To better understand how the chemical architecture of PEI influences complexation behavior to DNA cargo, we studied both LPEI and BPEI (Fig. 1). LPEI is a linear polymer that contains only secondary amines, while BPEI has a branched structure containing primary, secondary, and tertiary amines. These structural differences can potentially influence LPEI and BPEI complexation behavior and, consequently, their delivery mechanisms.

### 3.2. Morphologies of LPEI and BPEI polyplexes

**Fig. 2:**
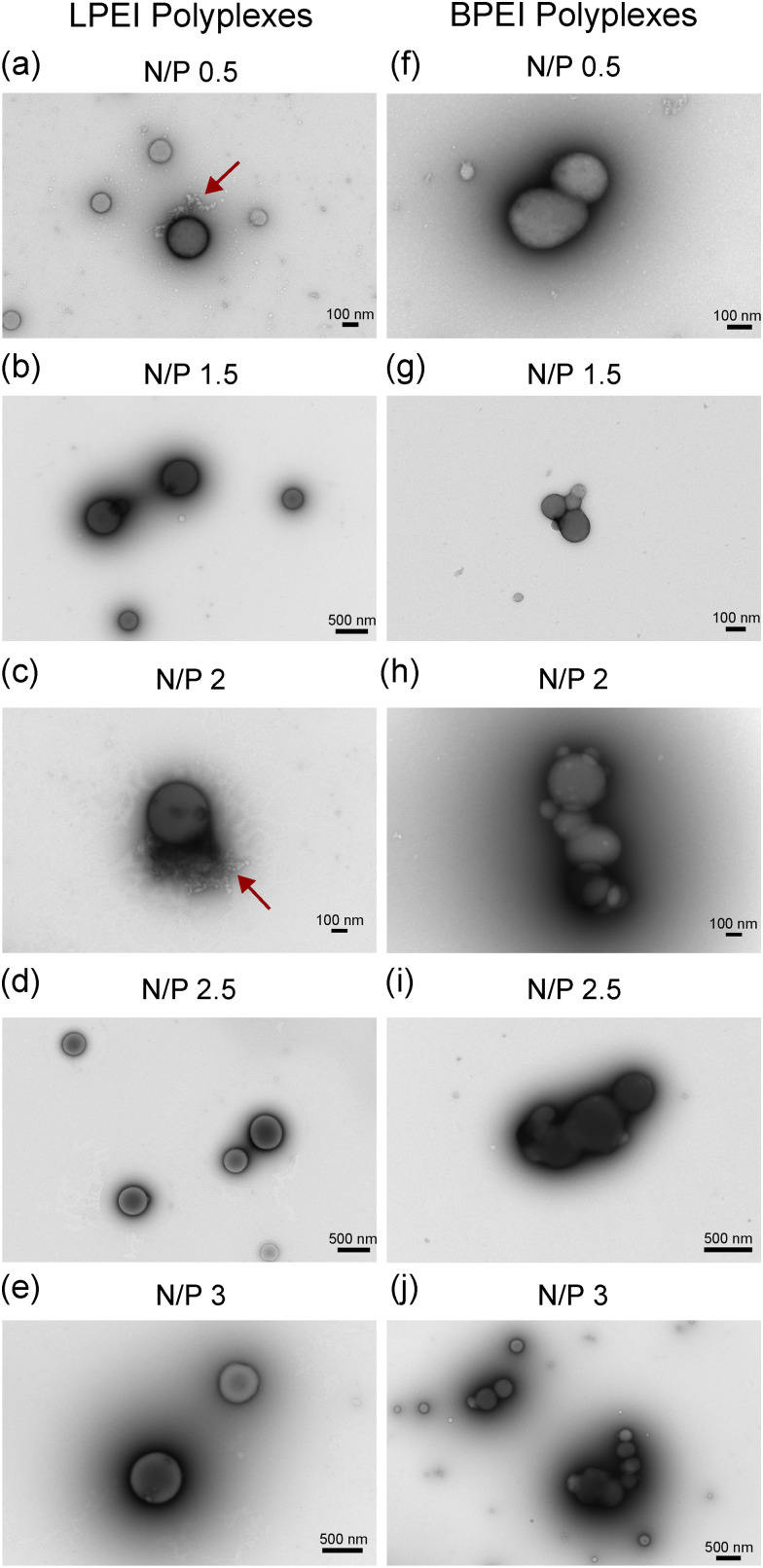
Representative TEM micrographs of LPEI and BPEI polyplexes at various N/P ratios: (a) and (f) N/P 0.5, (b) and (g) NP 1.5, (c) and (h) N/P 2, (d) and (i) N/P 2.5, (e) and (j) N/P 3. The scale bar is 100 nm for (a), (c), (f), (g) and (h). The scalebar is 500 nm for (b), (d), (e), (i) and (j). The red arrow shows the presence of DNA on the periphery of the polyplexes shown in (a) and (c).

To investigate how these chemical structural differences affect polyplex assembly, we first assessed the morphologies of LPEI and BPEI polyplexes. Fig. S1 (ESI†) shows the TEM images for samples containing only LPEI, BPEI, and DNA (controls), while Fig. 2 shows LPEI and BPEI polyplexes prepared at different N/P ratios. While LPEI, BPEI, and DNA exhibit fibrous morphologies (Fig. S1, ESI†), adding the polymers to DNA results in nanoparticles with spherical morphologies (Fig. 2), indicating the formation of polyplexes.^15,34–36^

Although both LPEI and BPEI form similar spherical structures, some distinct differences are observed. The images show that BPEI complexes exhibit significantly greater aggregation than LPEI complexes. Additionally, we observe that LPEI polyplexes sometimes exhibit less compact DNA structures, as indicated by the presence of free DNA on the periphery of the particles in some of the TEM images (Fig. 2a and c, red arrow). In contrast, we observe that BPEI tends to form polyplexes with more compact DNA structures that are not exposed on the exterior of the particles (Fig. 2f–j).

### 3.3. LPEI and BPEI exhibit different DNA binding behaviors

To further assess the binding behavior of LPEI and BPEI polyplexes, we investigated the binding affinities of these systems to DNA cargo (Fig. S2, ESI†). The fraction of DNA bound to polymer (*θ*) was determined using the equation:1
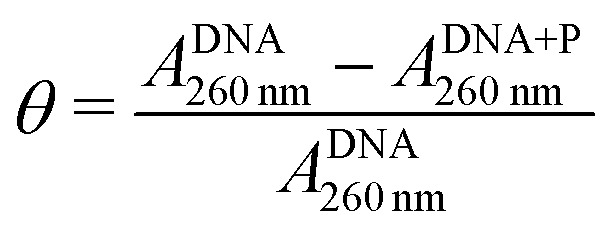
where *A*^DNA+P^_260nm_ and *A*^DNA^_260nm_ are the absorbances of DNA with and without polymer, respectively.

**Fig. 3:**
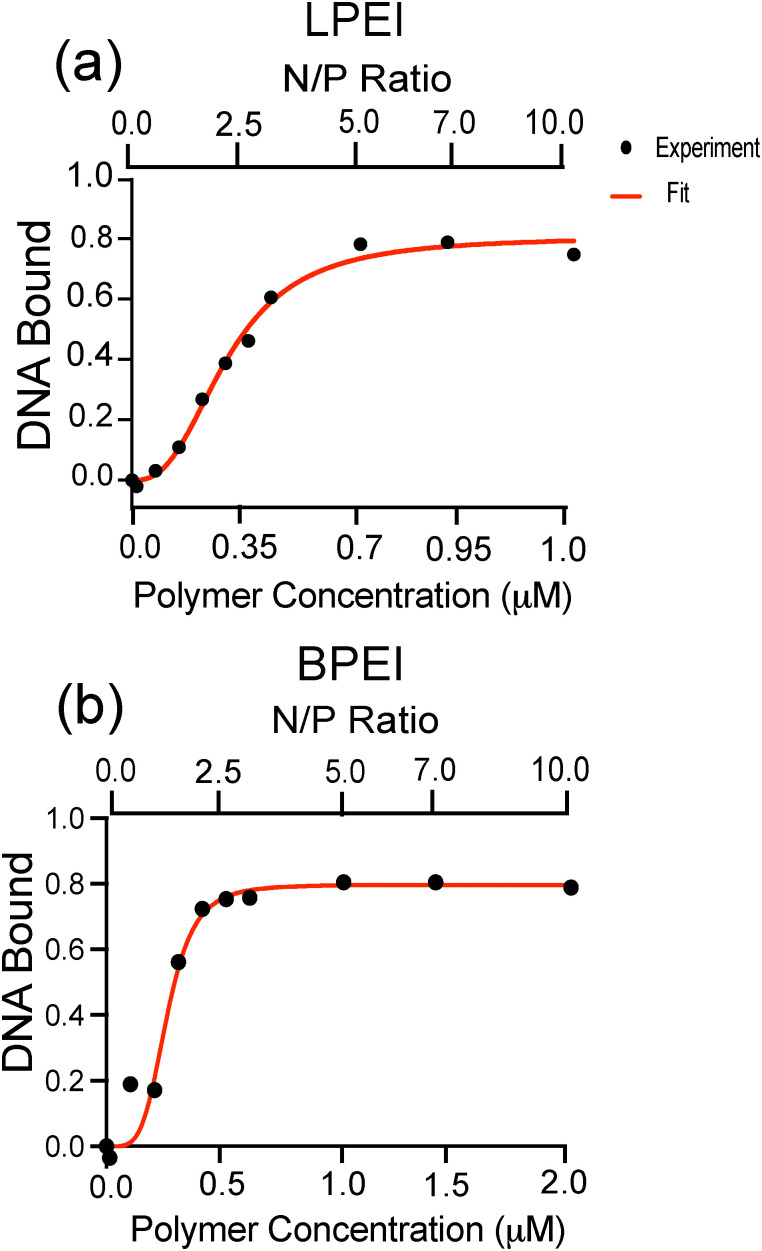
Binding curves of (a) LPEI (a) and (b) BPEI polyplexes with N/P ratios of 0, 0.1, 0.5, 1, 1.5, 2, 2.5, 3, 5, 7, 10. The DNA bound was calculated using the absorption values of DNA at 260 nm. The data was fit to a cooperative binding model using the Hill equation. The goodness-of-fit was assessed using the *R*^2^ value, which is 0.99 for LPEI and 0.97 for BPEI. Two replicates were measured and the standard deviation was calculated to be less than 2% of the mean for each data point shown.

The corresponding binding curves (Fig. 3) were fit to a cooperative binding model using the Hill equation:2
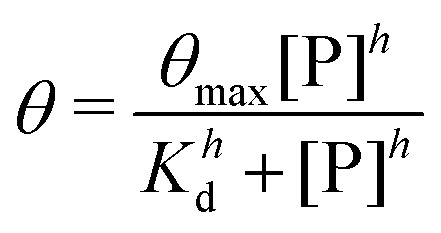
where *K*_d_ is the dissociation constant, [P] is the polymer concentration, *θ*_max_ is the maximum fraction of bound DNA, and *h* is the Hill coefficient.

Our binding affinity results suggest that LPEI and BPEI bind to DNA and form polyplexes through different mechanisms. DNA binding to both polymer systems saturate at approximately 80% (Fig. 3 and Table 1). Additionally, both LPEI and BPEI show positive binding cooperativity to DNA with similar affinities. However, BPEI has a higher Hill coefficient (4.4) than LPEI (2.6), indicating stronger cooperativity. This is likely due to its branched structure, which could enable additional binding modes provided by its primary, secondary, and tertiary amines.

**Table 1:** Summary of binding data for LPEI and BPEI polyplexes

Polymer	*K*_d_ (μM)	Hill coefficient (h)	*θ*_max_
LPEI	0.2879 (±0.01531)	2.661 (±0.3464)	0.8060 (±0.03125)
BPEI	0.2686 (±0.02395)	4.465 (±2.065)	0.7969 (±0.04546)

### 3.4. LPEI and BPEI interact with and package DNA cargo differently

**Fig. 4:**
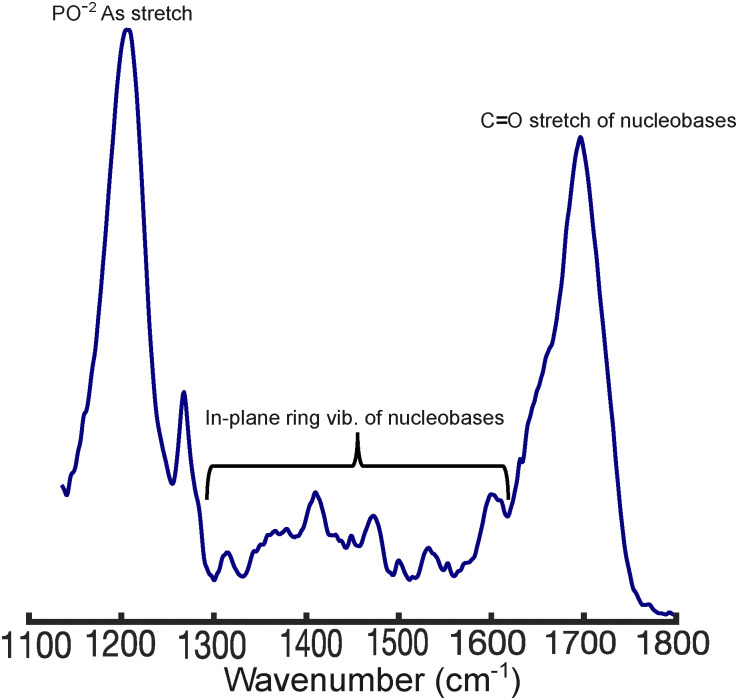
FTIR spectrum of 1 mM DNA in phosphate buffer, pH = 5.0. The spectrum was blank subtracted, baselined, and normalized to the total integrated area.

While the binding studies show key differences in polyplex formation, IR spectroscopy provides molecular-level insights into their interactions. To investigate this, we measured the FTIR spectra of free DNA, LPEI, and BPEI, as well as DNA in the presence of these polymers at N/P ratios ranging from 0–3. The spectra of LPEI and BPEI (Fig. S3, ESI†) are relatively weak compared to DNA, showing bands around ∼1450 cm^−1^ (CH_2_ deformation) and 1620 cm^−1^ (NH_2_ scissoring).^37^ In contrast, DNA (Fig. 4) possesses strong bands at ∼1222 cm^−1^ (PO^−2^ asymmetric stretching) and ∼ 1680 (C=O stretching of thymine and guanine). Additional weak bands that derive from in-plane ring stretching modes of the nucleobases appear between 1250–1600 cm^−1^.^38–42^

**Fig. 5:**
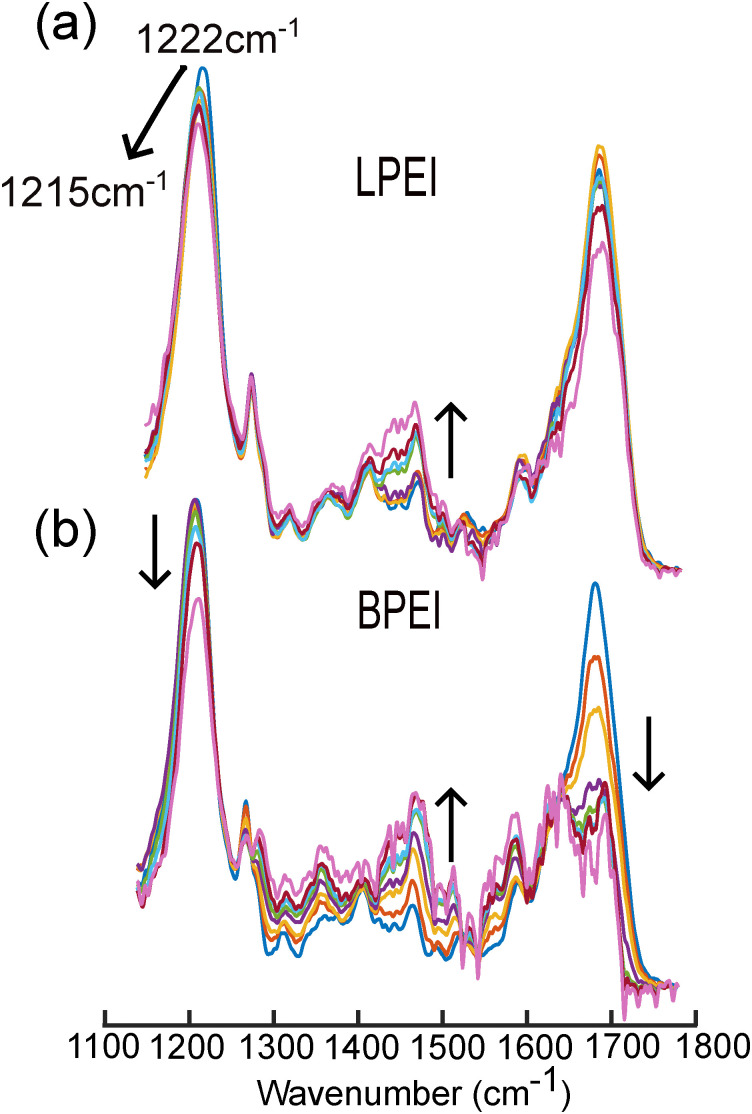
FTIR spectra of DNA in the presence of (a) LPEI and (b) BPEI in phosphate buffer, pH 5.0. The black arrow in each set of spectra represents the spectral changes that occur from N/P ratios of 0, 0.25, 0.5, 1, 1.5, 2, 2.5, 3. All the spectra were blank subtracted, baselined, and normalized to the total integrated area.

Significant spectral changes are observed in the DNA bands upon the addition of LPEI and BPEI (Fig. 5). For example, the 1222 cm^−1^ band of DNA decreases in intensity and downshifts by ∼7 cm^−1^ as the N/P ratio of LPEI increases (Fig. 5a). Additionally, although they overlap partially with the ∼1450 cm^−1^ band of the polymers, the nucleobase ring stretching bands between 1400–1500 cm^−1^ increase in intensity as the N/P ratio increases. The 1680 cm^−1^ band also exhibits intensity changes, although no clear trend is observed with increasing N/P ratio. These spectral changes are similar to those observed in polyplexes prepared from transfection-grade PEI (Fig. S4, ESI†), indicating that it binds DNA similarly to LPEI.

In contrast, BPEI binding (Fig. 5b) results in complex changes in the DNA bands between ∼1300–1500 cm^−1^, which generally increase in intensity as a function of N/P ratio. In addition, pronounced intensity decreases for both the 1222 cm^−1^ and 1680 cm^−1^ DNA bands are observed as the N/P ratio increases. However, unlike LPEI, binding of BPEI to DNA does not result in a frequency shift of the PO^−2^ asymmetric stretching band at 1222 cm^−1^.

These spectral changes suggest that LPEI and BPEI bind DNA and package it into polyplexes through distinct mechanisms that involve interactions with both the phosphate backbone and nucleobases. For example, the frequency shift and intensity changes in the 1222 cm^−1^ band indicate that LPEI primarily binds DNA through strong electrostatic interactions with the phosphate backbone. The downshift of this band to 1215 cm^−1^ at high N/P ratios further suggests that LPEI binding to the phosphate backbone induces a conformational transition in DNA from B-form to Z-form.^43,44^ We observed similar structural changes in DNA when complexed to transfection-grade PEI (Fig. S4, ESI†).

In contrast, the absence of a frequency shift in this band for BPEI suggests weaker binding to the phosphate backbone that does not alter the backbone conformation of DNA. Instead, the significant decrease in intensity of this band suggests that the primary amines of BPEI likely form hydrogen bonds with the phosphate oxygen atoms of DNA in addition to electrostatic interactions. Additionally, the substantial intensity changes observed in the nucleobase bands between 1300–1500 cm^−1^ as well as the splitting of the 1680 cm^−1^ band indicate that BPEI also intercalates into the DNA duplex, most likely through hydrogen bonding interactions with nucleobases. The concomitant increase in the intensity of the bands between 1300–1500 cm^−1^ indicates that BPEI interactions with the nucleobases disrupts their native π-stacking interactions in the DNA.

An alternative hypothesis is that the spectral changes in the nucleobase bands at 1680 cm^−1^ and between 1300–1500 cm^−1^ could also occur due to DNA condensation upon polyplex formation. Condensation of DNA could result in increased rigidity of the nucleobases. However, the base-specific intensity variations unique to BPEI, as opposed to LPEI, point more convincingly toward stronger nucleobase-polymer interactions in the case of BPEI compared to LPEI.

### 3.5. The presence of NaCl confirms the existence of two binding modes for LPEI and BPEI polyplexes

To validate our spectral interpretation, we examined LPEI and BPEI polyplex formation in the presence of NaCl. We hypothesized that Na^+^ and Cl^−^ could screen electrostatic interactions and thereby inhibit polymer binding to the DNA phosphate backbone. If LPEI primarily interacts with DNA *via* electrostatic forces, its binding should be more sensitive to salt screening effects than BPEI, which our data suggests preferentially interacts with nucleobases.

**Fig. 6:**
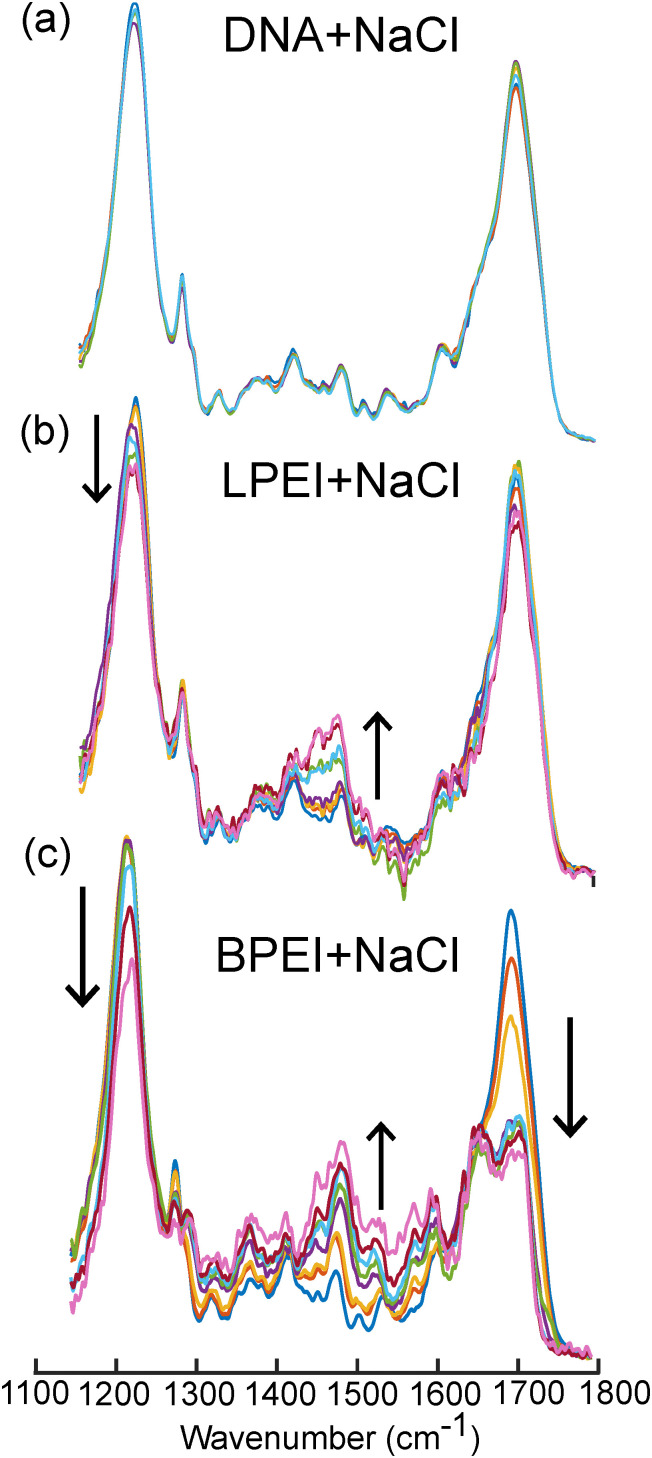
FTIR spectra of DNA in the presence of (a) 50–250 mM NaCl, (b) LPEI and NaCl (250 mM), and (c) BPEI (250 mM). The black arrows in (b) and (c) indicate spectral trends going from N/P ratios of 0, 0.25, 0.5, 1, 1.5, 2, 2.5, 3. All the spectra were blank subtracted, baselined, and normalized to the total integrated area.

Fig. 6 and Fig. S5 (ESI†) show the IR spectra of DNA with LPEI and BPEI at varying N/P ratios and NaCl concentrations. The spectra of DNA in the presence of NaCl (Fig. 6a) confirm that Na^+^ ions do not significantly bind DNA on their own. However, when introduced to polymer–DNA solutions (Fig. 6b and c), clear differences can be seen in the complexation behavior of LPEI and BPEI.

**Fig. 7:**
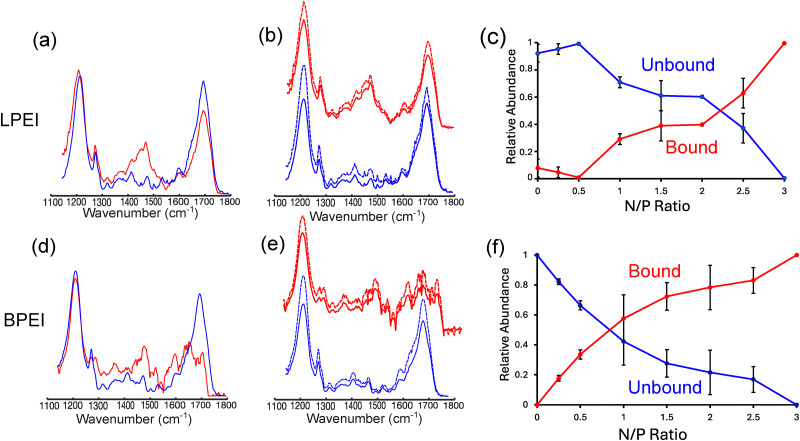
Basis spectra derived from MCR-ALS analysis for bound (red, solid line) and unbound (blue, solid line) DNA in experiments involving binding to (a) LPEI and (d) BPEI. These spectra match the experimental bound (red, dashed line) and unbound (blue, dashed line) (b) and (e) DNA spectra. Panels (c) and (f) show concentration profiles for the fraction of DNA bound and unbound to (c) LPEI and (f) BPEI as a function of N/P ratio at 0 mM NaCl. The error bars in the concentration profiles represent the uncertainties that are derived from the average of 2 replicates.

To better understand these differences, we constructed polyplex probability diagrams for LPEI and BPEI as a function of both N/P ratio and NaCl concentration. These probability diagrams help visualize the chemical conditions that favor polyplex formation. Using multivariate curve resolution-alternating least-squares (MCR-ALS) analysis^33^ (see ESI† for details), we decomposed the experimental spectra of LPEI and BPEI polyplexes to extract two basis spectra, one representing polymer-bound DNA and the other representing unbound DNA (Fig. 7a and d). The extracted basis spectra closely match the experimental spectra of bound and unbound DNA (Fig. 7b and e), confirming the accuracy of the decomposition. Using these basis set spectra, we determined the fraction of bound and unbound DNA under different NaCl concentration conditions and polymer N/P ratios (Fig. S5, ESI†). We accomplished this by modeling each experimental spectrum as a linear combination of the bound and unbound DNA basis spectra. Using this methodology, we estimate that the average error in calculating the fraction bound and unbound DNA from the FTIR spectra is ∼6% for LPEI and ∼7% for BPEI complexes (Table S1, ESI†).

From the extracted concentration profiles, we then calculated the probability of DNA binding to LPEI and BPEI (*P*_b_) (Fig. 7c and f and Fig. S7, ESI†):3
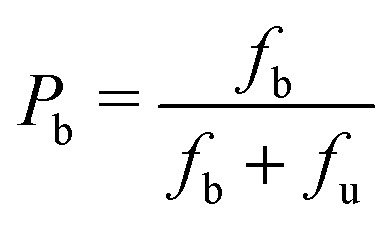
where *f*_b_ and *f*_u_ are the relative fractions of bound and unbound DNA obtained from the MCR-ALS fitting analysis. The probabilities obtained from eqn (3) were then used to construct the probability diagrams for LPEI and BPEI polyplexes.

**Fig. 8:**
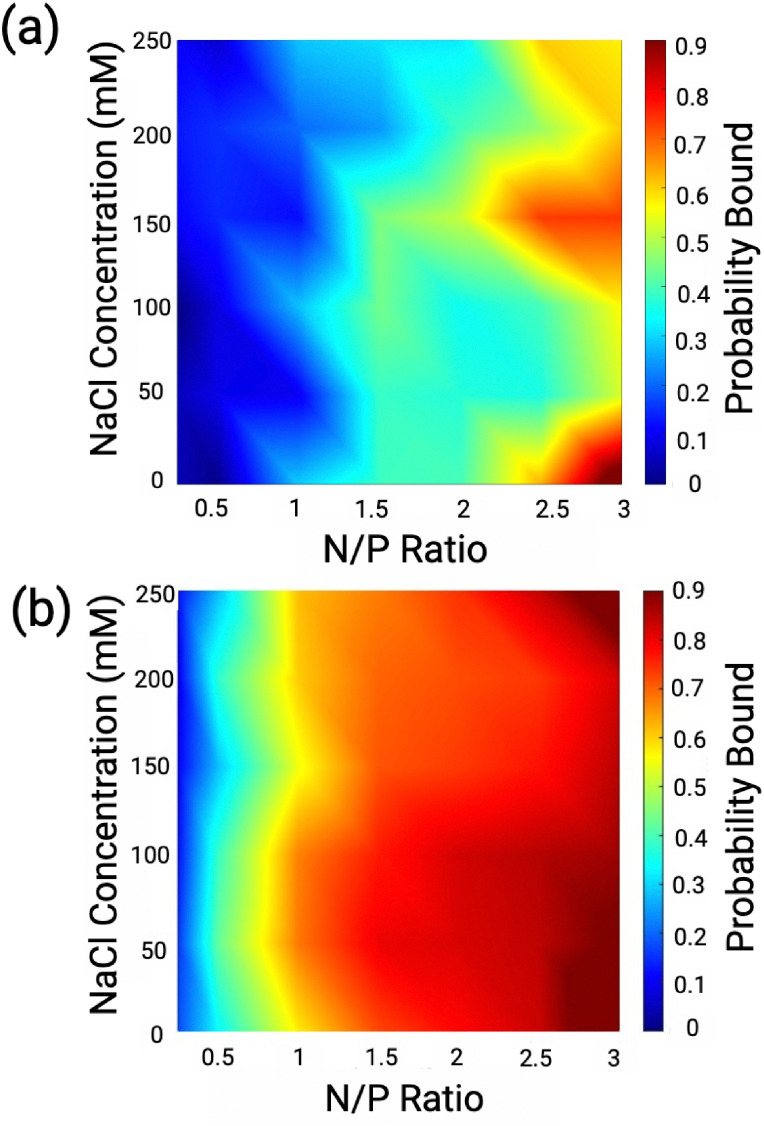
Probability diagrams showing the probability of DNA bound to (a) LPEI and (b) BPEI as a function of N/P ratio and NaCl concentration.

The resulting probability diagrams (Fig. 8) show that NaCl impacts LPEI and BPEI complexation to DNA differently. NaCl significantly reduces LPEI binding to DNA by up to ∼70% (Fig. 8a). In contrast, NaCl has a significantly smaller effect on BPEI binding to DNA, except at high concentrations (200 and 250 mM) (Fig. 8b), where binding is reduced by ∼20–30%. The significant susceptibility of LPEI binding to NaCl validates our hypothesis that it preferentially binds DNA through interactions with the phosphate backbone rather than through hydrogen bonding interactions with the nucleobases. In contrast, the insensitivity of BPEI binding to NaCl supports the notion that it interacts with DNA cargo primarily through hydrogen bonding interactions with the nucleobases.

### 3.6. Proposed mechanism of LPEI and BPEI binding to DNA

In this study, we have employed TEM, UV, and IR absorption spectroscopy to investigate the DNA complexation mechanisms of LPEI and BPEI involved in polyplex formation. Our key findings include:

1. TEM images show that LPEI and BPEI complexes both form spherical morphologies. BPEI complexes appeared more aggregated and compacted than LPEI.

2. Both LPEI and BPEI exhibit positive cooperativity binding to DNA with BPEI showing a slightly higher cooperativity than LPEI.

3. IR measurements reveal two distinct molecular binding modes to DNA for LPEI and BPEI. The IR spectra show that LPEI preferentially binds DNA through electrostatic interactions with the phosphate backbone, while BPEI binds DNA primarily through interactions with the DNA nucleobases.

**Fig. 9:**
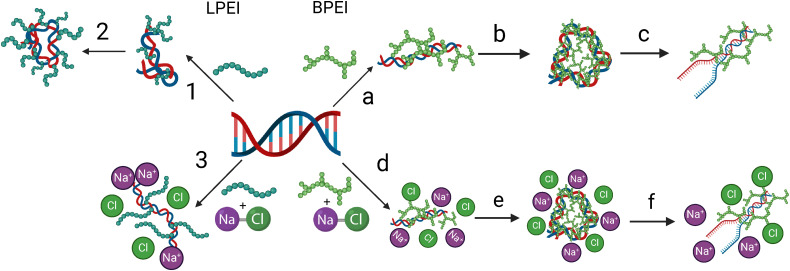
Suggested mechanism for LPEI and BPEI binding to DNA and polyplex formation. For LPEI, step (1) represents the electrostatic binding between the positive secondary amine groups and the phosphate backbone of DNA while step (2) represents DNA condensation. For BPEI, step (a) represents the hydrogen bonding and electrostatic interactions between the primary amines and the oxygen on the phosphate backbone of the DNA. BPEI also intercalates into the DNA bases in this step. In step (b) BPEI causes DNA condensation. The final (step c) shows a possible disruption to the DNA bases. The mechanism also shows that the addition of NaCl inhibits LPEI binding to DNA (step 3) while it does not impact the binding of BPEI (step d–f). Created with Biorender.

Taken together, we present a binding model for LPEI and BPEI polyplexes in Fig. 9. Our model suggests that LPEI binds electrostatically to the phosphate backbone of DNA with a weaker binding to the nucleobases (Fig. 9, step 1). This binding causes DNA structural rearrangements from B-form to Z-form and condensation (Fig. 9, step 2). The addition of NaCl inhibits the binding of LPEI to the phosphate backbone, thereby making binding to the DNA nucleobases preferable (Fig. 9, step 3). In contrast, BPEI binds DNA through weaker interactions to the phosphate backbone, as well as hydrogen bonding interactions with the nucleobases (Fig. 9, step a). This multi-modal binding causes efficient condensation of the DNA, which subsequently contributes to the disruption of native π-stacking interactions of the nucleobases (Fig. 9, steps b and c). The addition of NaCl does not significantly impact binding of BPEI to DNA (Fig. 9, steps d–f).

## 4. Conclusion

Developing effective polymers that can deliver multiple types of cargo is essential for advancing polymer-based gene therapies. This can be achieved by designing polymers with multiple binding mechanisms while optimizing cargo release. PEI-based delivery systems are among these and can be optimized to efficiently deliver plasmid DNA, mRNA, and CRISPR-Cas9 technology.^14,27,45^ PEI is traditionally thought to interact with nucleic acids through electrostatic forces, but our findings show that multiple binding modes play a key role in DNA condensation. Recognizing and using these distinct binding mechanisms provides a foundation for engineering more versatile PEI-based platforms and formulations that improve DNA release, accommodation of other cargo types, and enhance gene delivery.

## Author contributions

Rusul Mustafa: conceptualization (equal); formal analysis (equal); investigation (equal); methodology (equal); writing – original draft (lead); writing – review & editing (equal). Danielle Diorio: formal analysis (supporting); investigation (supporting); methodology (supporting); writing – review & editing (supporting). Madeline Harper: investigation (supporting); writing – review & editing (supporting). David Punihaole: conceptualization (equal); formal analysis (equal); funding acquisition (lead); methodology (equal); project administration (lead); resources (lead); supervision (lead); writing – original draft (supporting); writing – review & editing (equal).

## Data availability

The data supporting this article have been included as part of the ESI.†

## Conflicts of interest

There are no conflicts to declare.

## Supplementary Material

SM-021-D5SM00213C-s001

SM-021-D5SM00213C-s002
