# Supplementary figures and images for: Revealing two distinct molecular binding modes in polyethyleneimine–DNA polyplexes using infrared spectroscopy
Source: Soft Matter. 2025 Apr 26;21(21):4192–200. doi: 10.1039/d5sm00213c (PMC12053835; doi:10.1039/d5sm00213c)

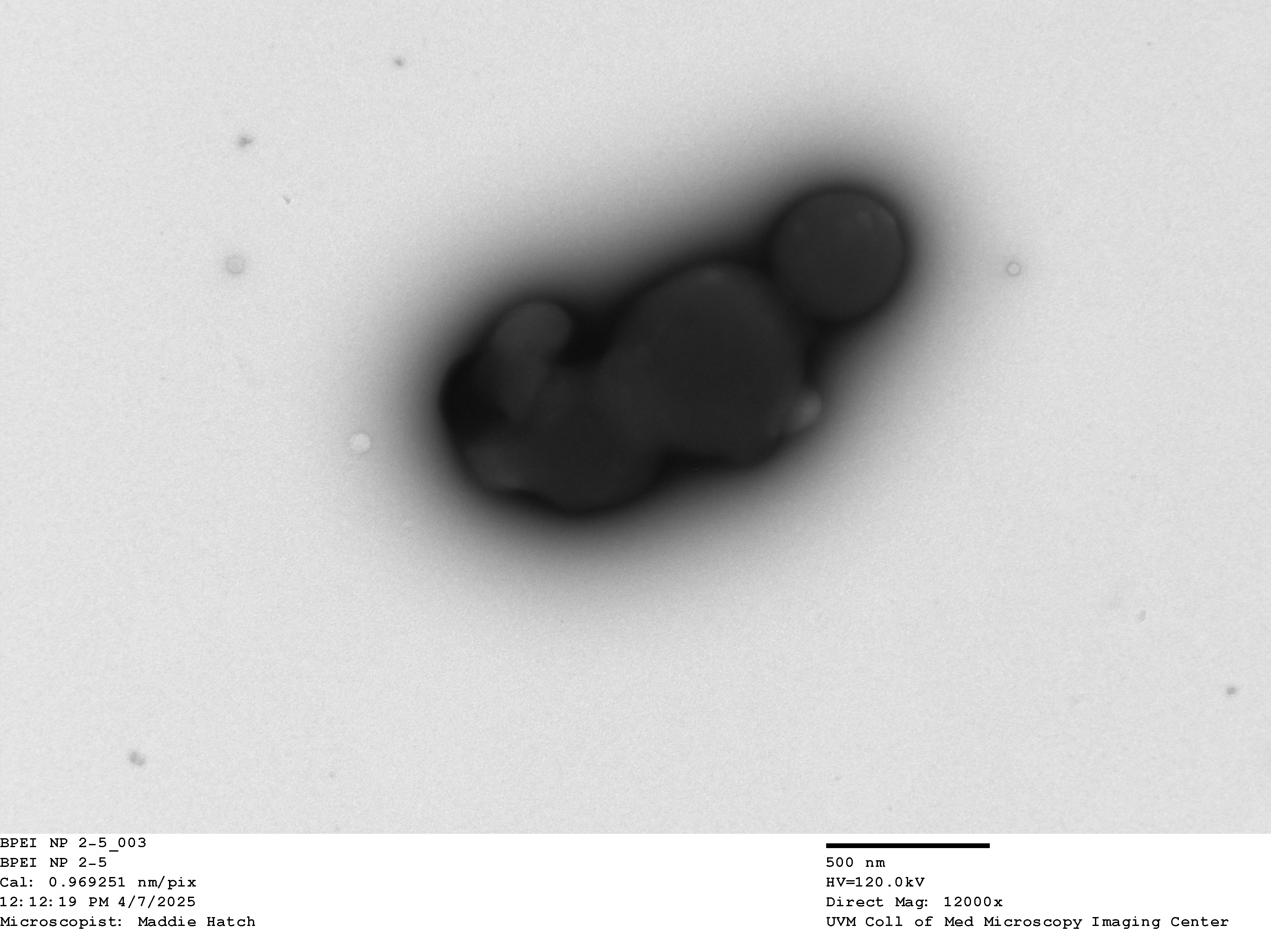

Supplement: SM-021-D5SM00213C-s001 [file SM-021-D5SM00213C-s001.zip › bpei np 2-5_003 copy.tif]

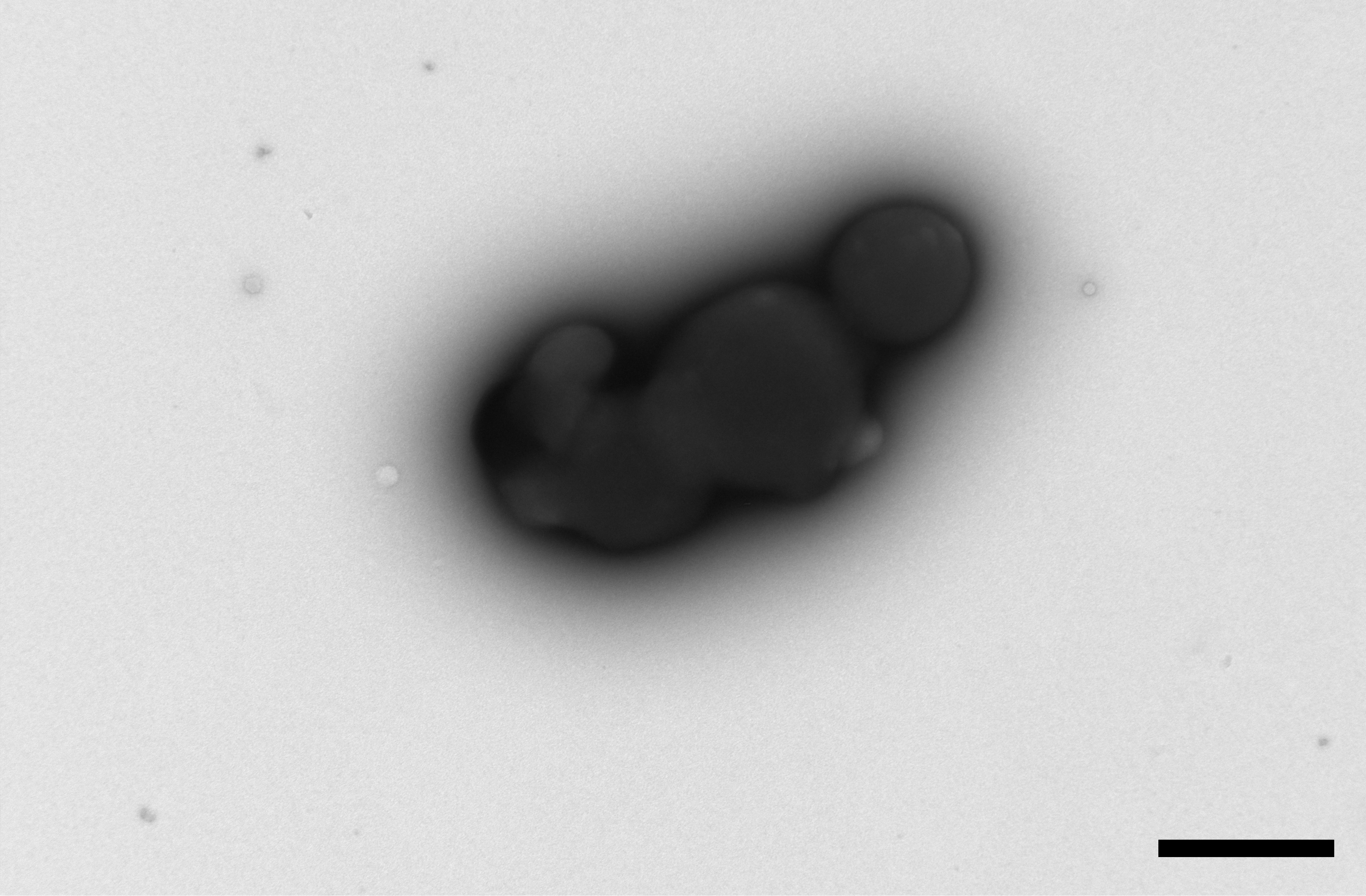

Supplement: SM-021-D5SM00213C-s001 [file SM-021-D5SM00213C-s001.zip › bpei np 2-5_003 copy-500nmscalebar.jpg]

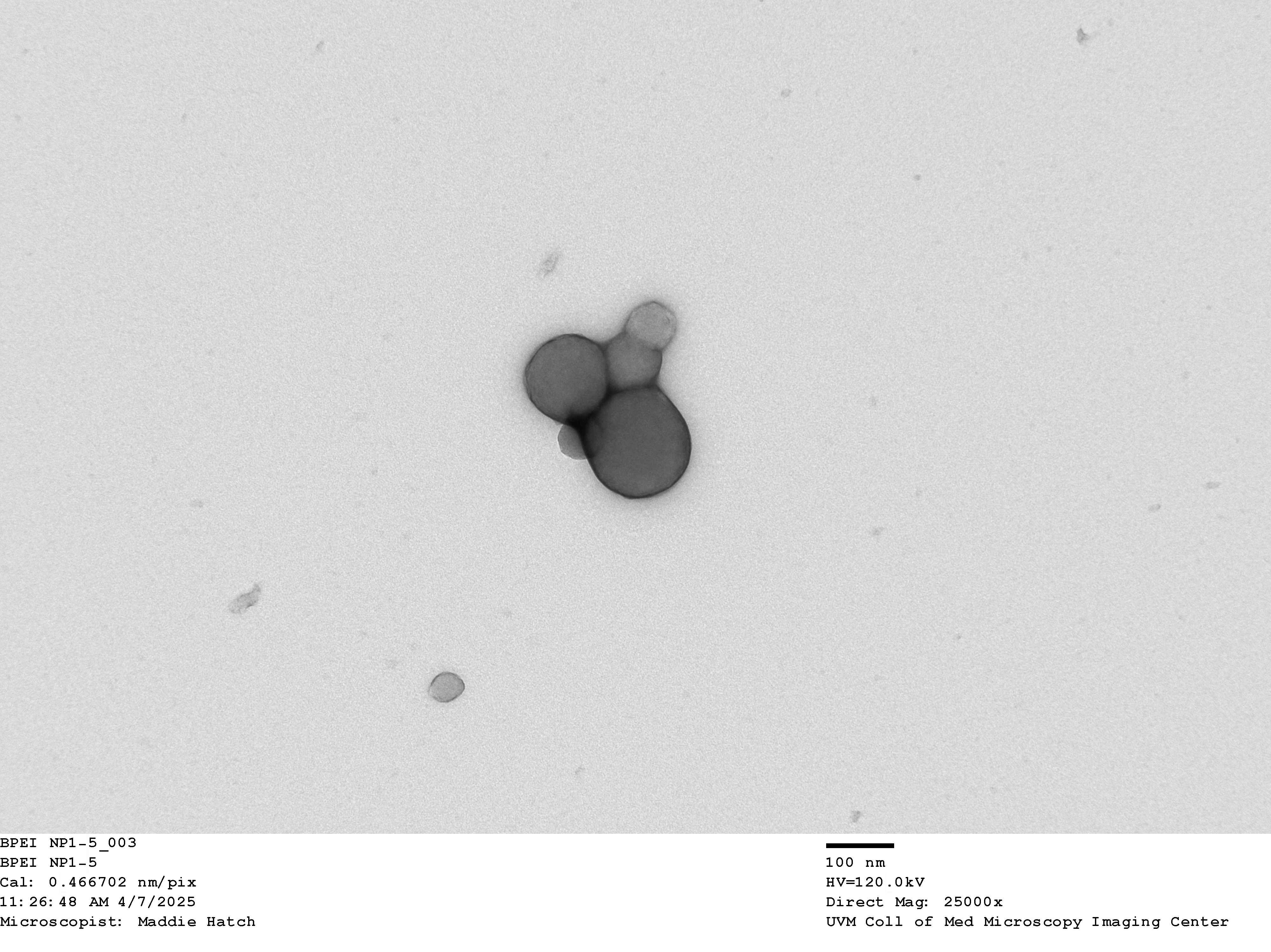

Supplement: SM-021-D5SM00213C-s001 [file SM-021-D5SM00213C-s001.zip › bpei np1-5_003 copy.tif]

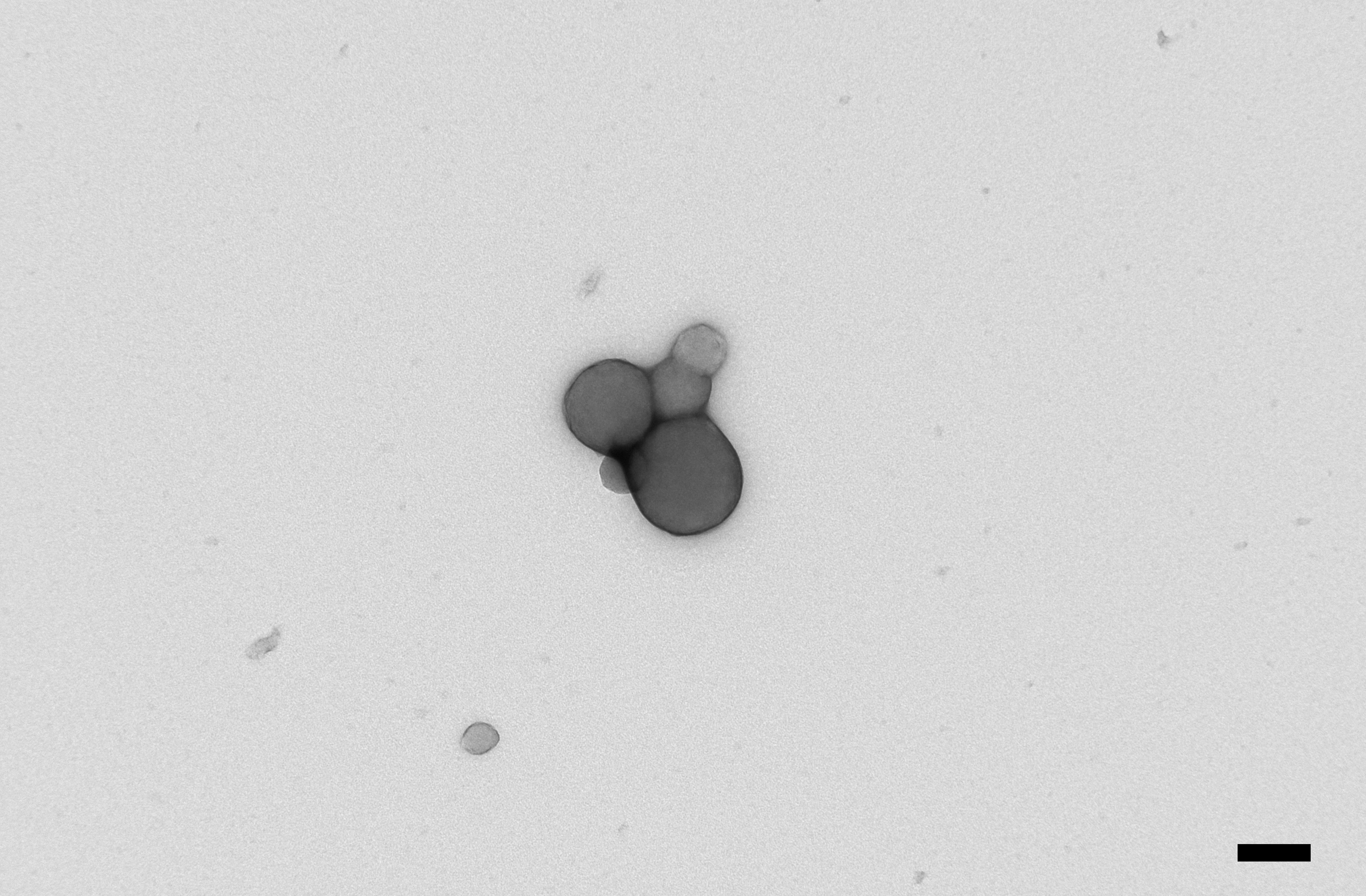

Supplement: SM-021-D5SM00213C-s001 [file SM-021-D5SM00213C-s001.zip › bpei np1-5_003 copy-100nmscalebar.jpg]

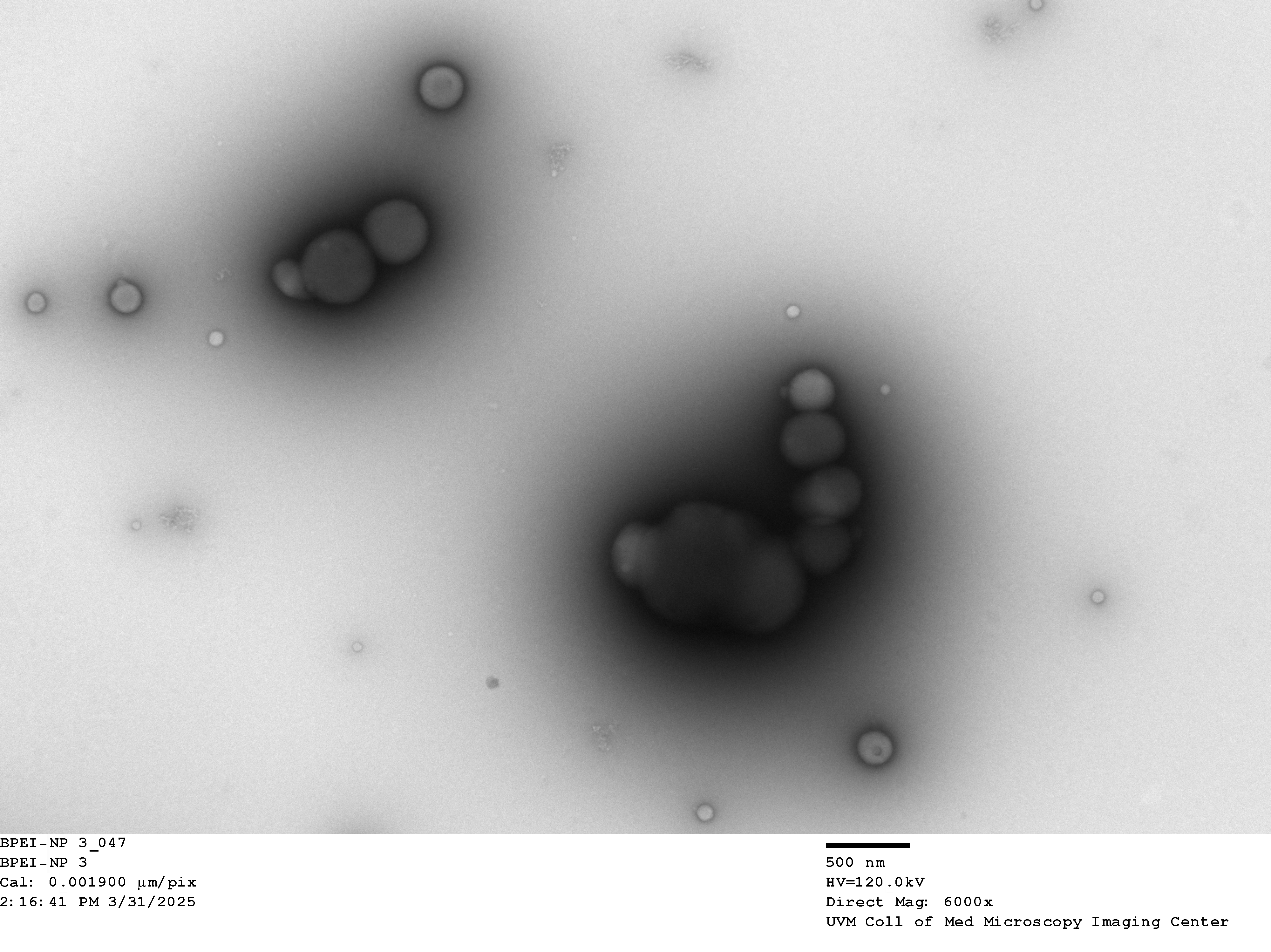

Supplement: SM-021-D5SM00213C-s001 [file SM-021-D5SM00213C-s001.zip › bpei-np 3_047 copy.tif]

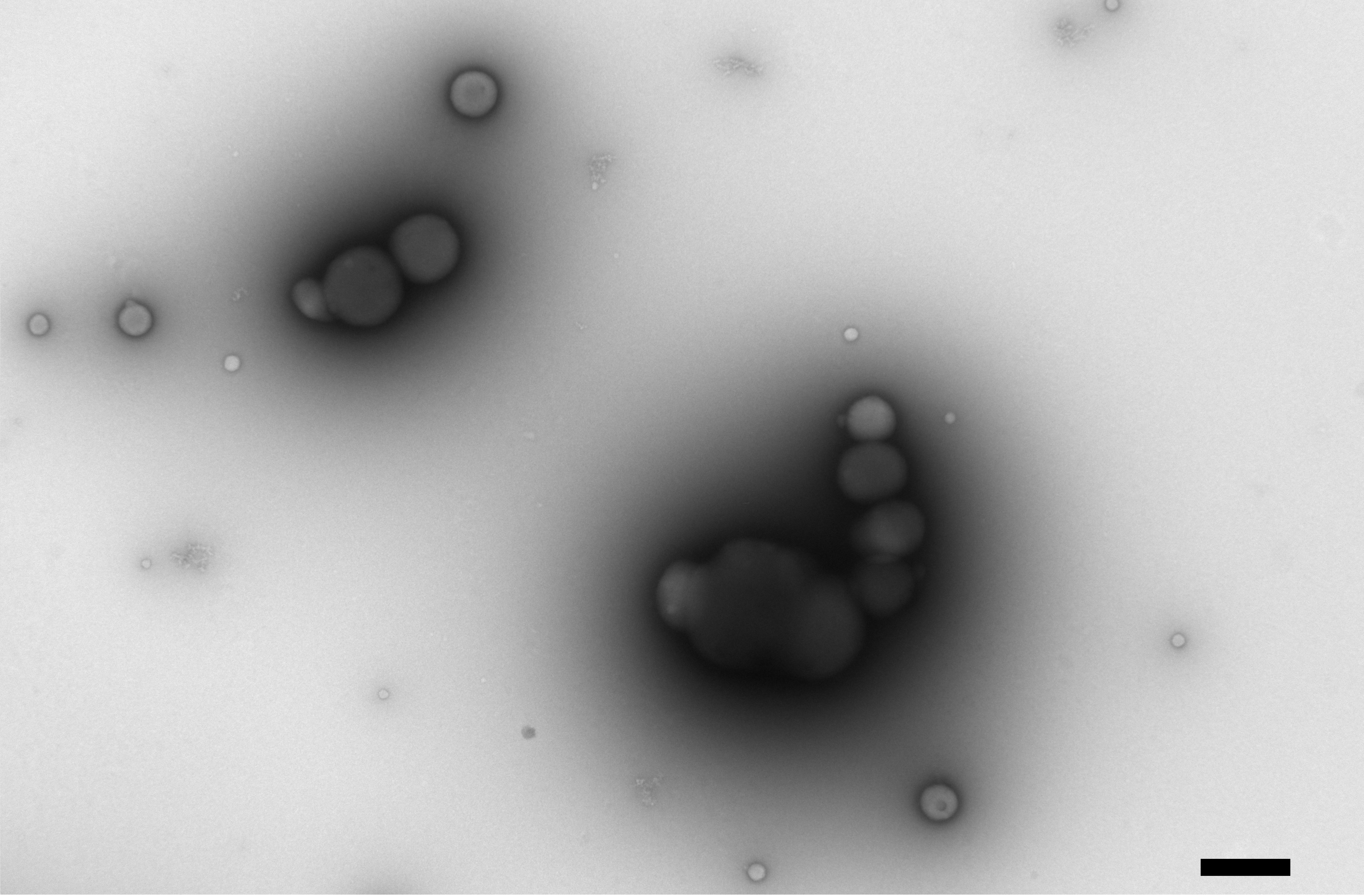

Supplement: SM-021-D5SM00213C-s001 [file SM-021-D5SM00213C-s001.zip › bpei-np 3_047 copy-500nmscalebar.jpg]

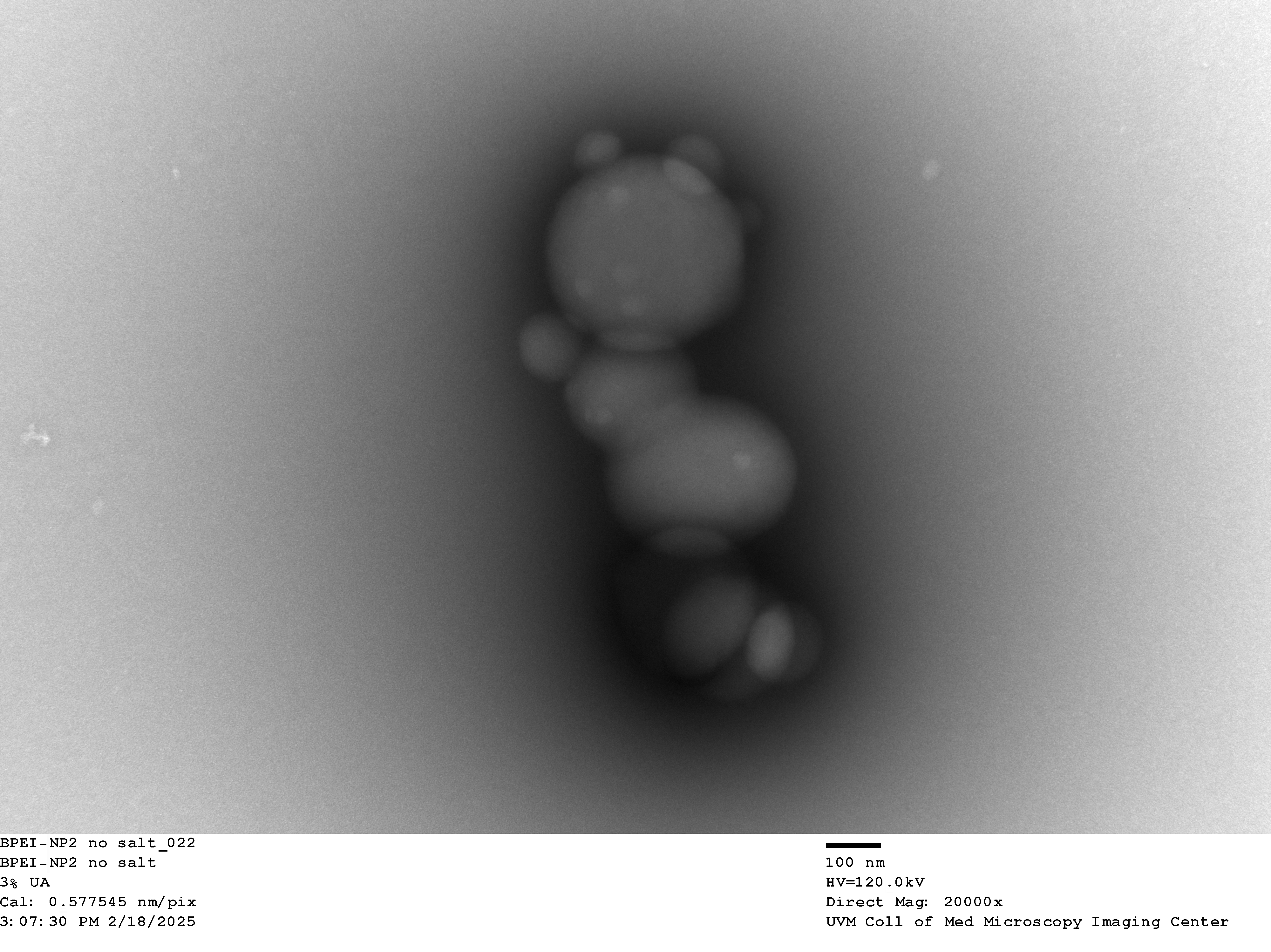

Supplement: SM-021-D5SM00213C-s001 [file SM-021-D5SM00213C-s001.zip › bpei-np2 no salt_022 copy.tif]

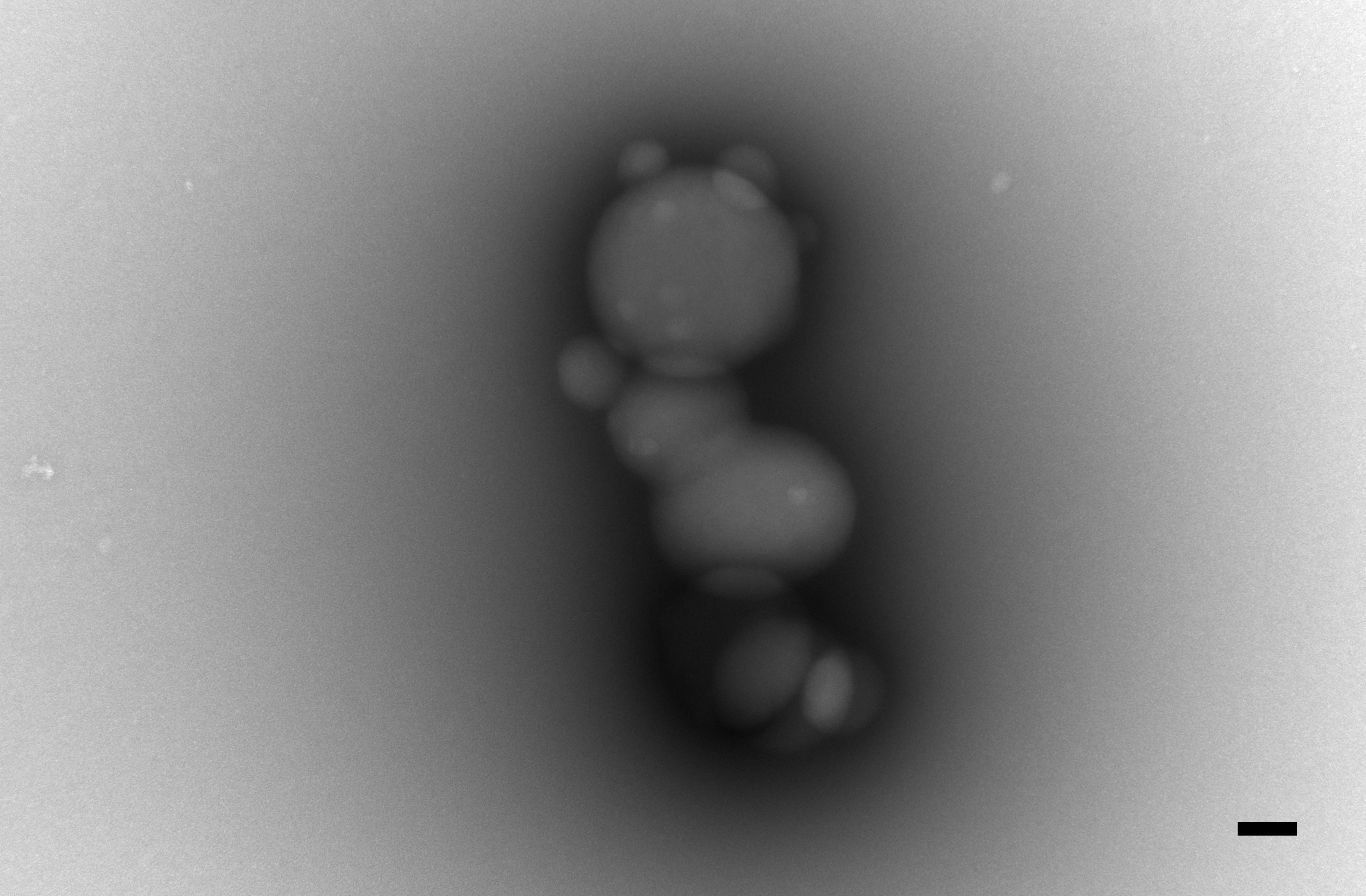

Supplement: SM-021-D5SM00213C-s001 [file SM-021-D5SM00213C-s001.zip › bpei-np2 no salt_022 copy-100nmscalebar-crop.jpg]

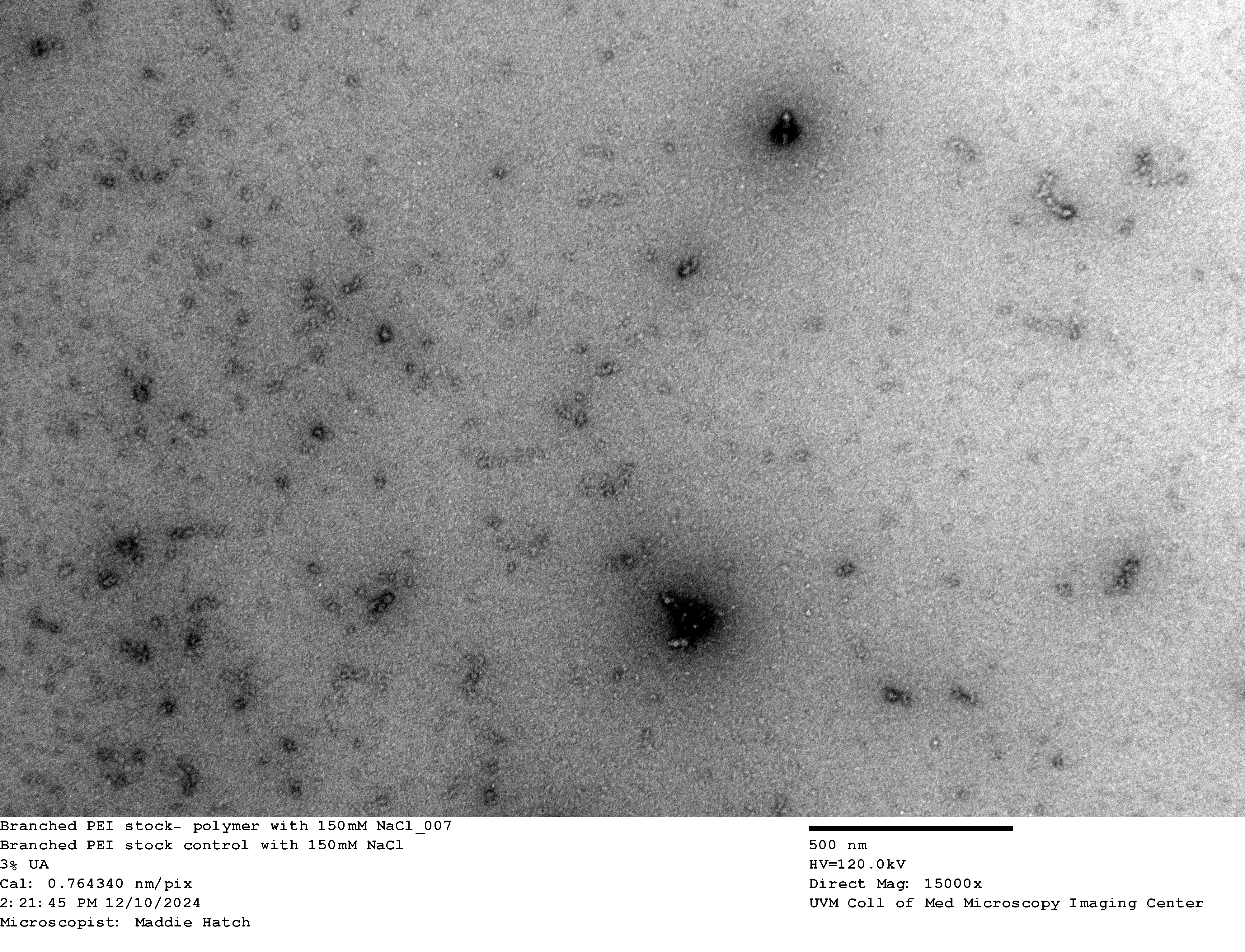

Supplement: SM-021-D5SM00213C-s001 [file SM-021-D5SM00213C-s001.zip › branched pei stock- polymer with 150mm nacl_007.tif]

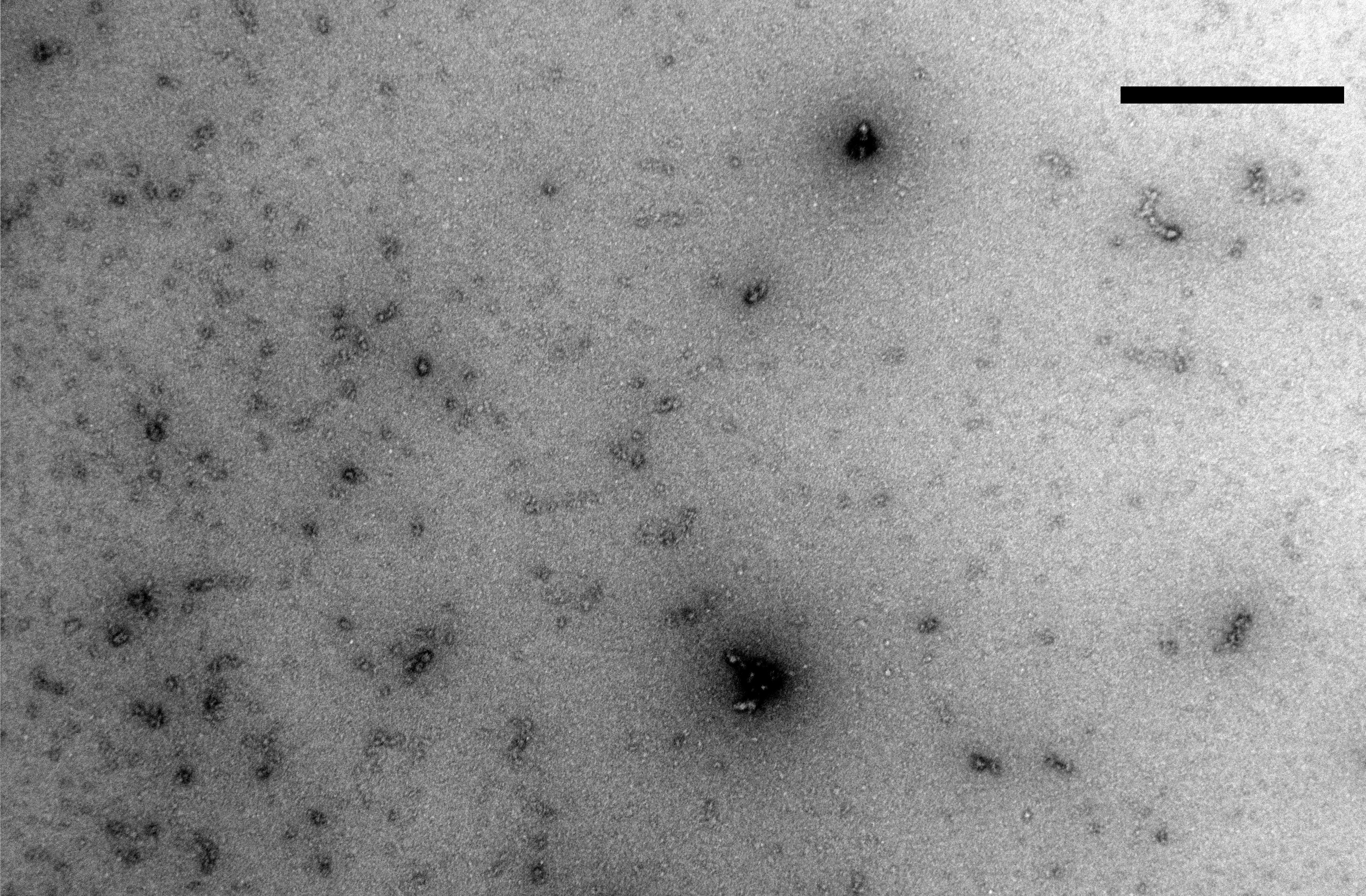

Supplement: SM-021-D5SM00213C-s001 [file SM-021-D5SM00213C-s001.zip › branched pei stock- polymer with 150mm nacl_007-500nmscalebar-2.jpg]

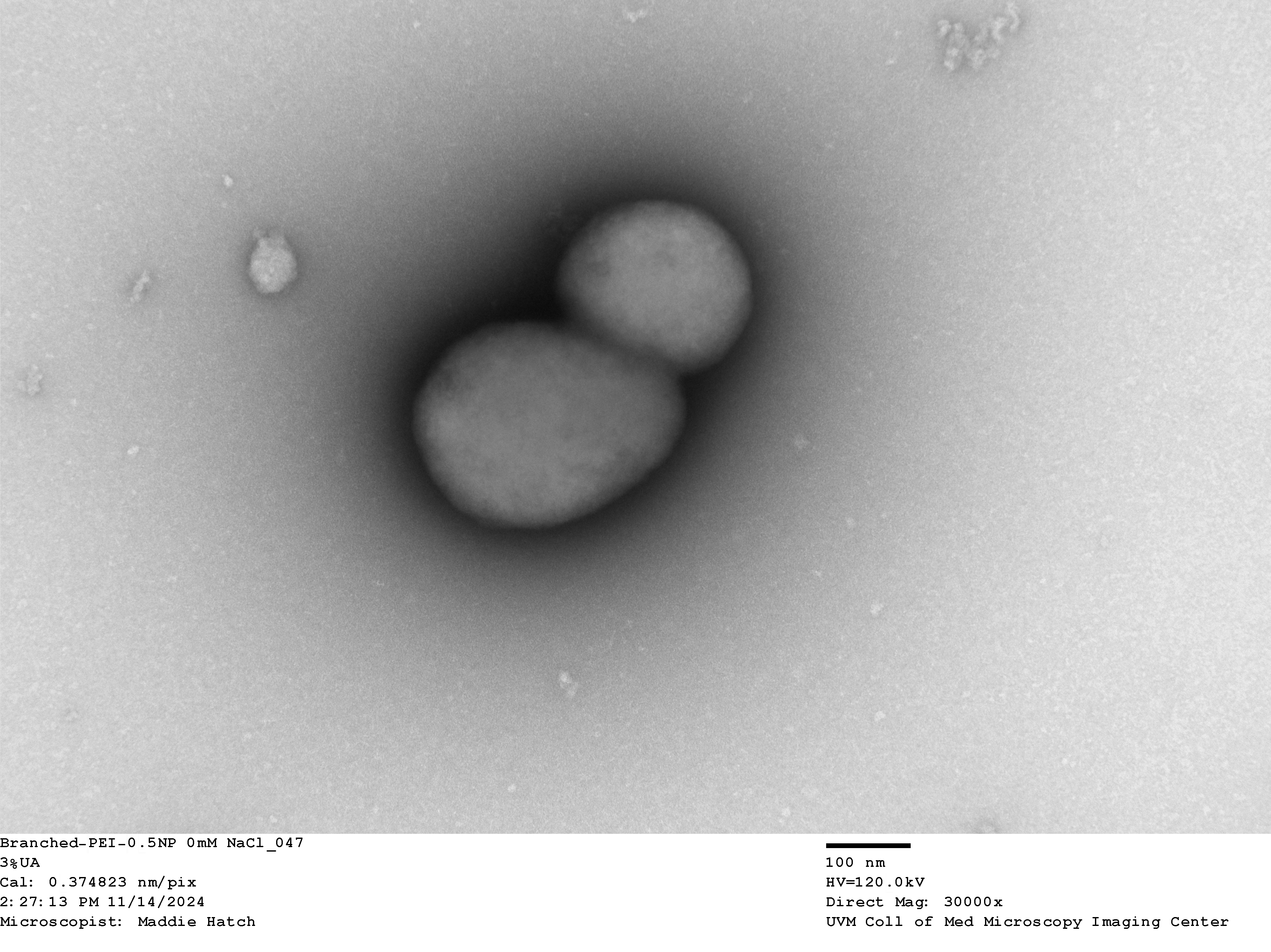

Supplement: SM-021-D5SM00213C-s001 [file SM-021-D5SM00213C-s001.zip › branched-pei-0.5np 0mm nacl_047 copy.tif]

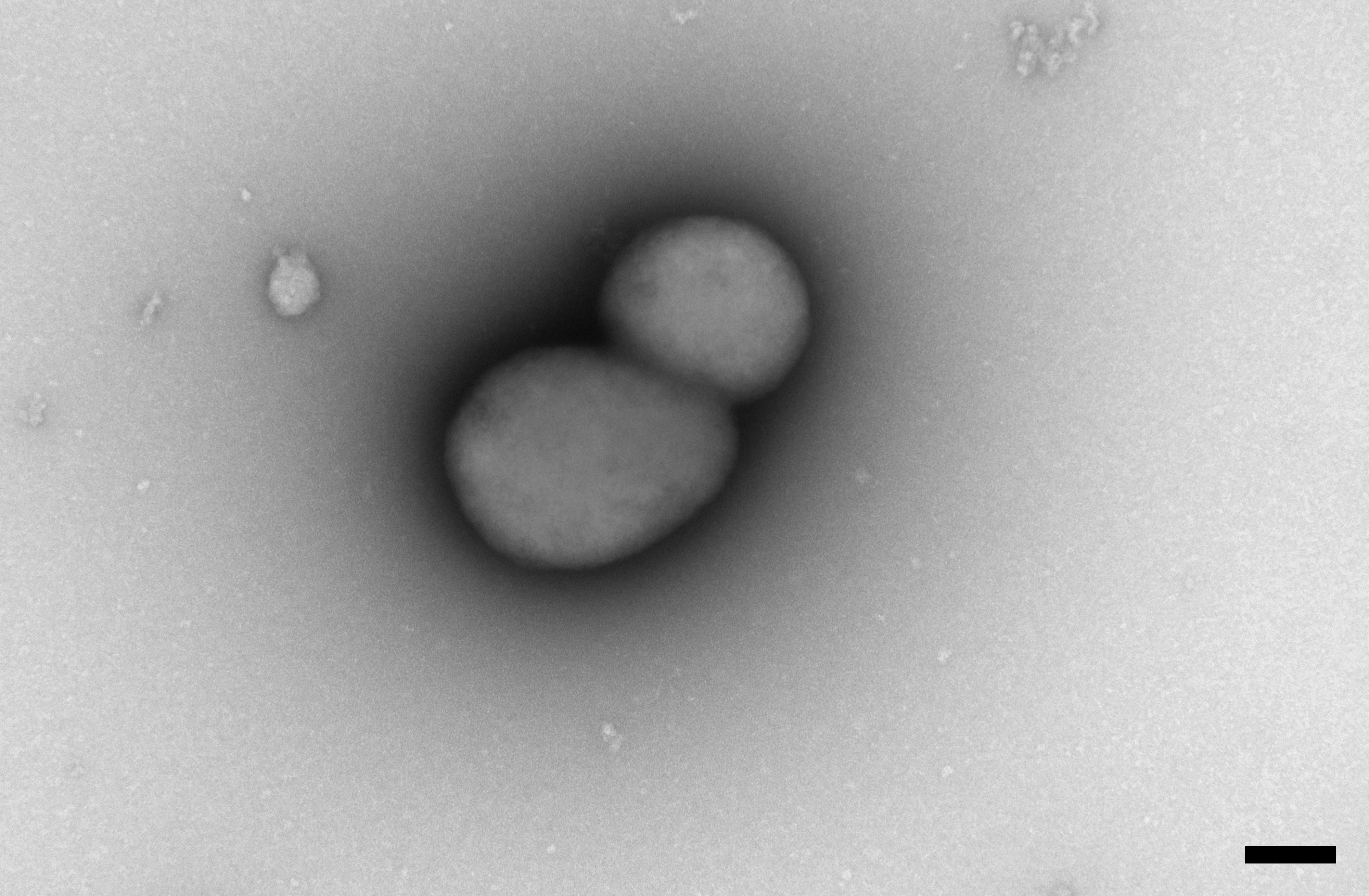

Supplement: SM-021-D5SM00213C-s001 [file SM-021-D5SM00213C-s001.zip › branched-pei-0.5np 0mm nacl_047 copy-100nmscalebar.jpg]

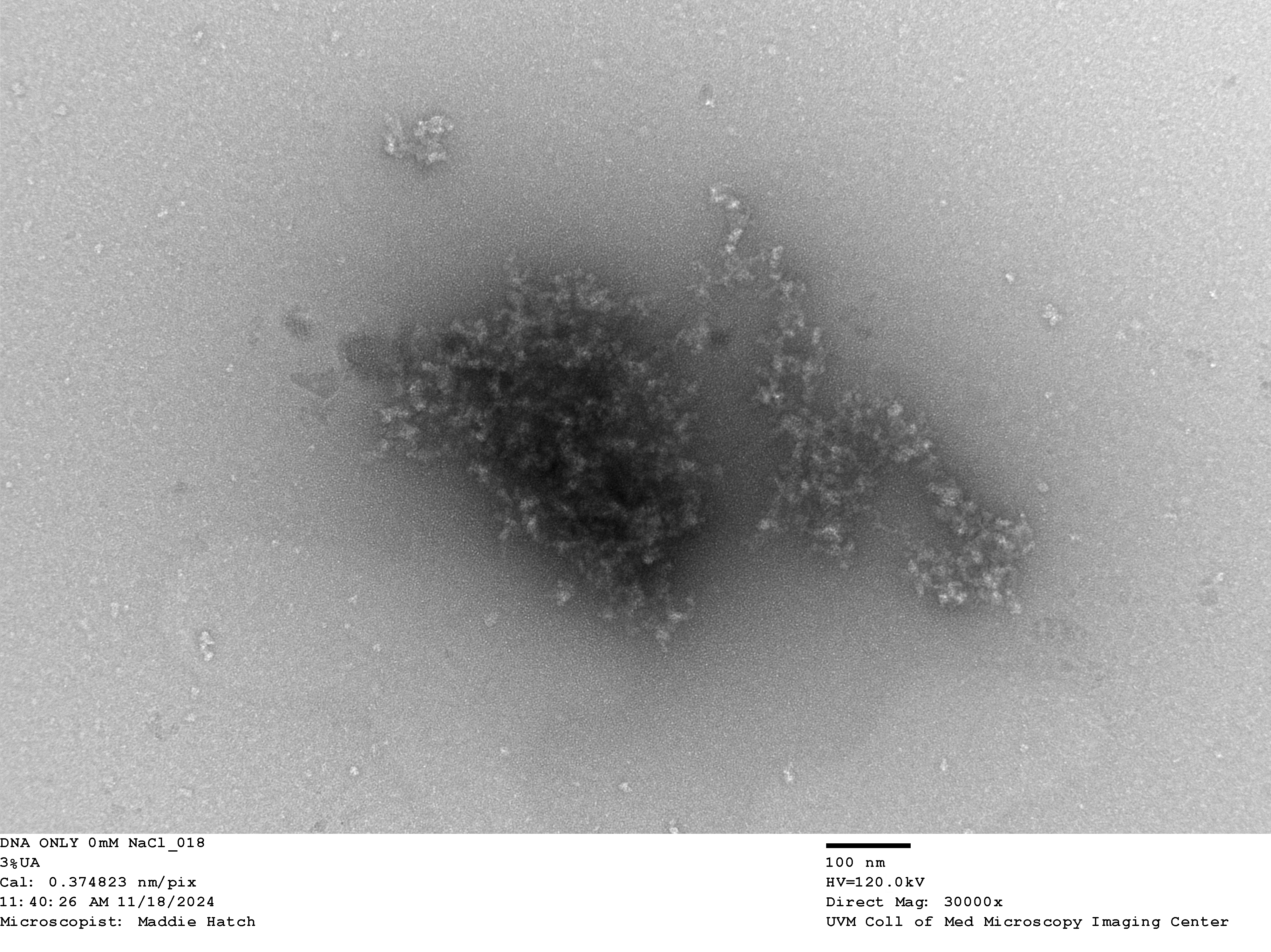

Supplement: SM-021-D5SM00213C-s001 [file SM-021-D5SM00213C-s001.zip › dna only 0mm nacl_018.tif]

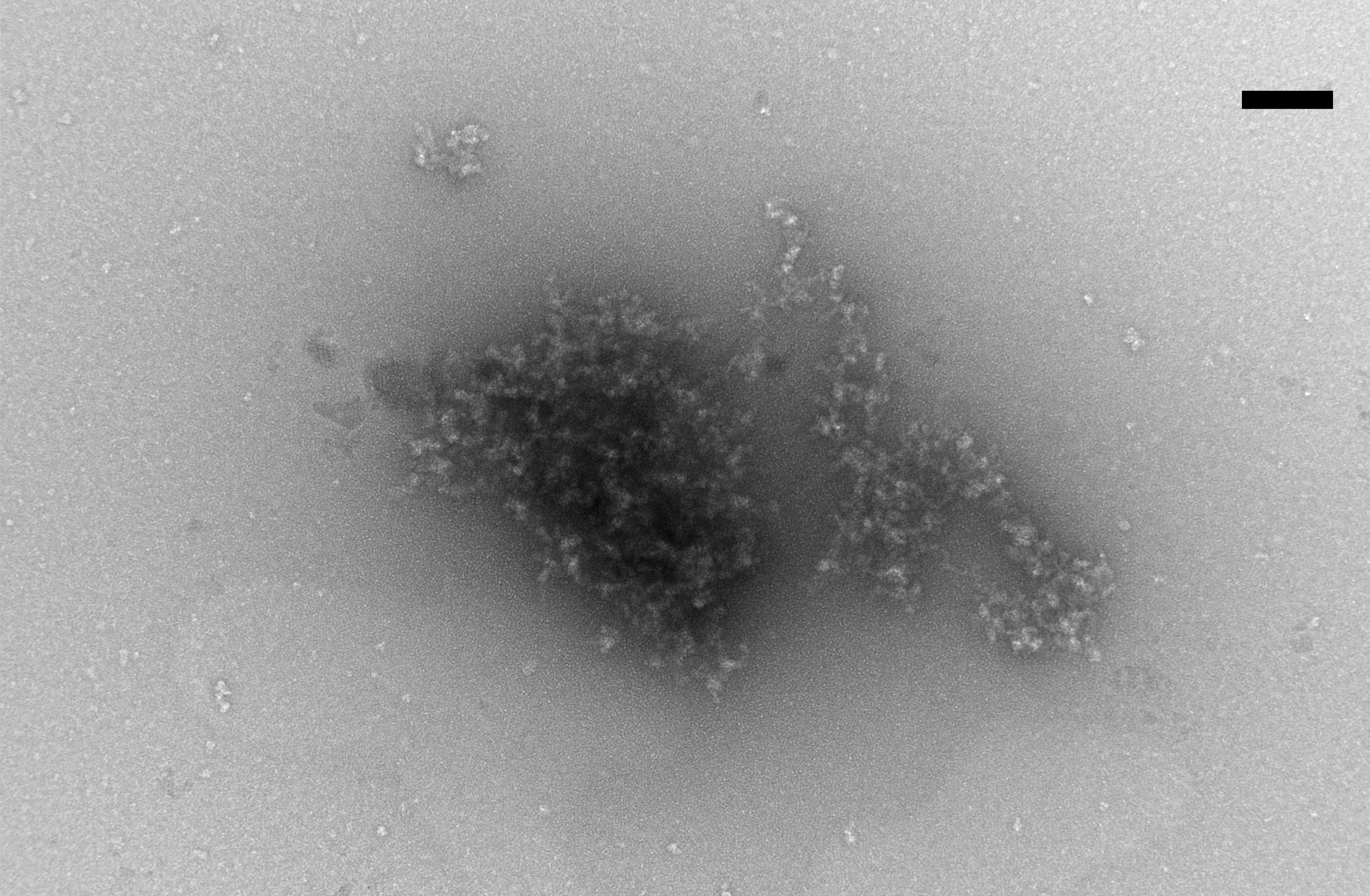

Supplement: SM-021-D5SM00213C-s001 [file SM-021-D5SM00213C-s001.zip › dna only 0mm nacl_018-scalebar100nm-crop2.jpg]

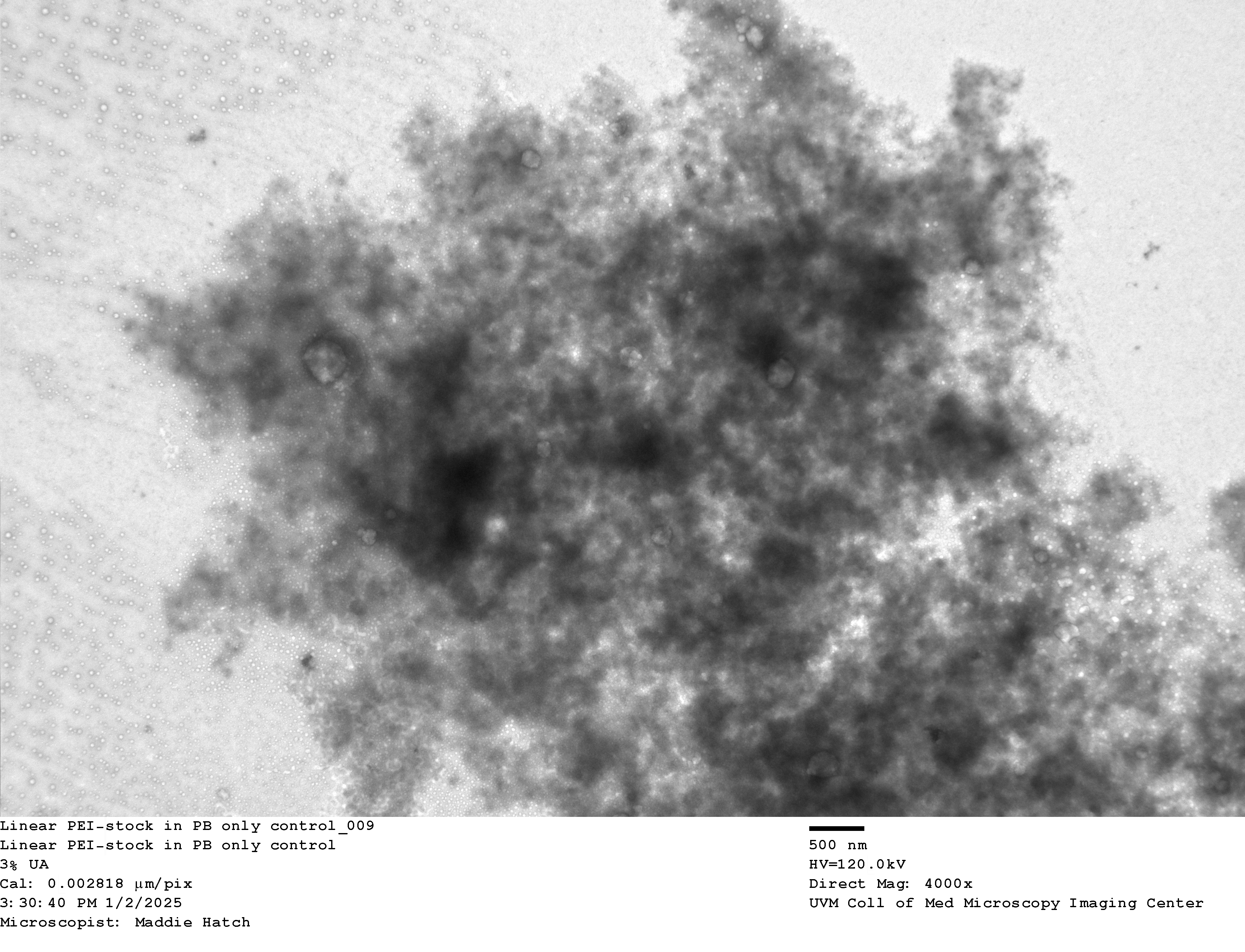

Supplement: SM-021-D5SM00213C-s001 [file SM-021-D5SM00213C-s001.zip › linear pei-stock in pb only control_009.tif]

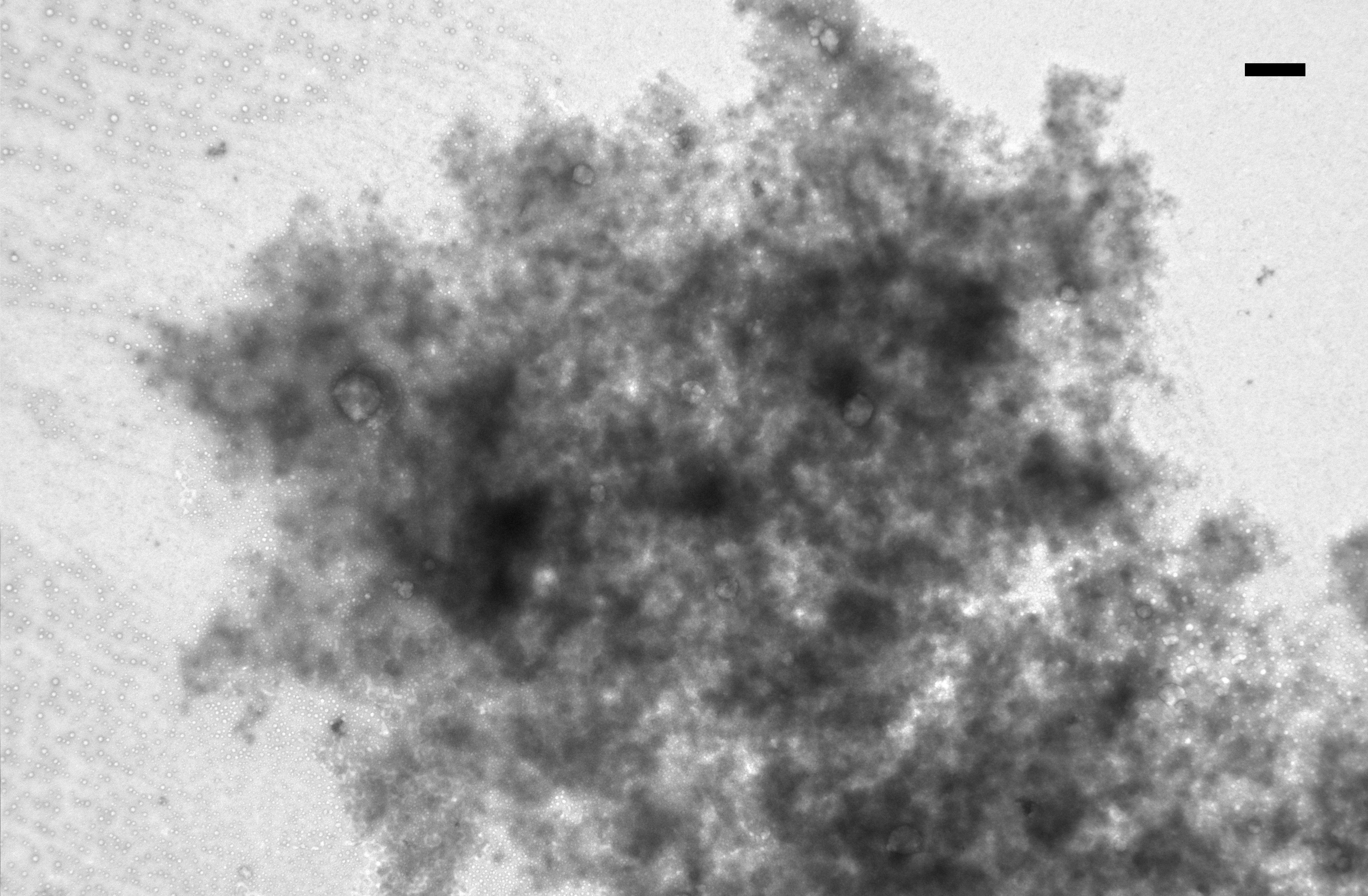

Supplement: SM-021-D5SM00213C-s001 [file SM-021-D5SM00213C-s001.zip › linear pei-stock in pb only control_009-500nmscalebar-crop.jpg]

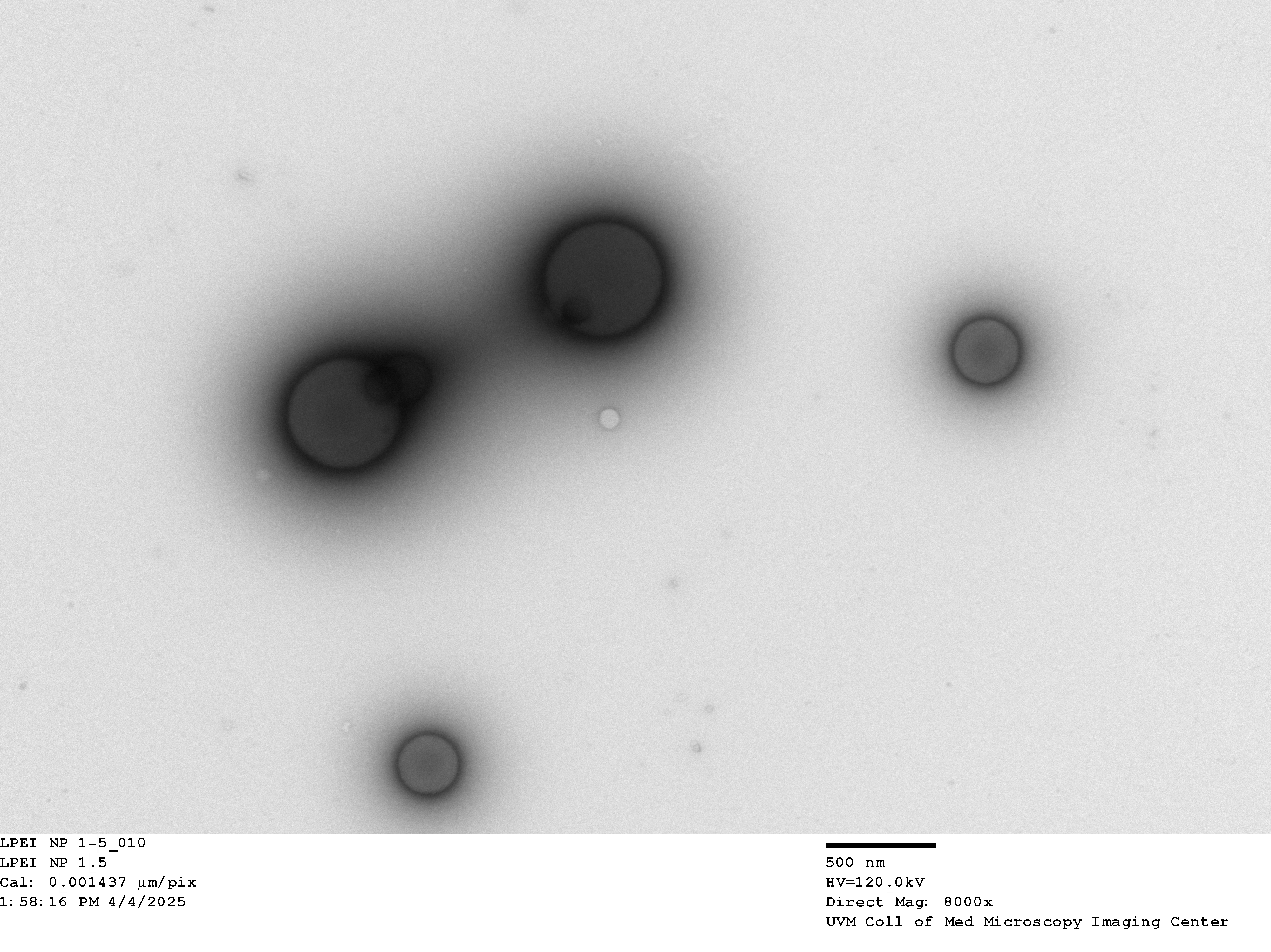

Supplement: SM-021-D5SM00213C-s001 [file SM-021-D5SM00213C-s001.zip › lpei np 1-5_010 copy.tif]

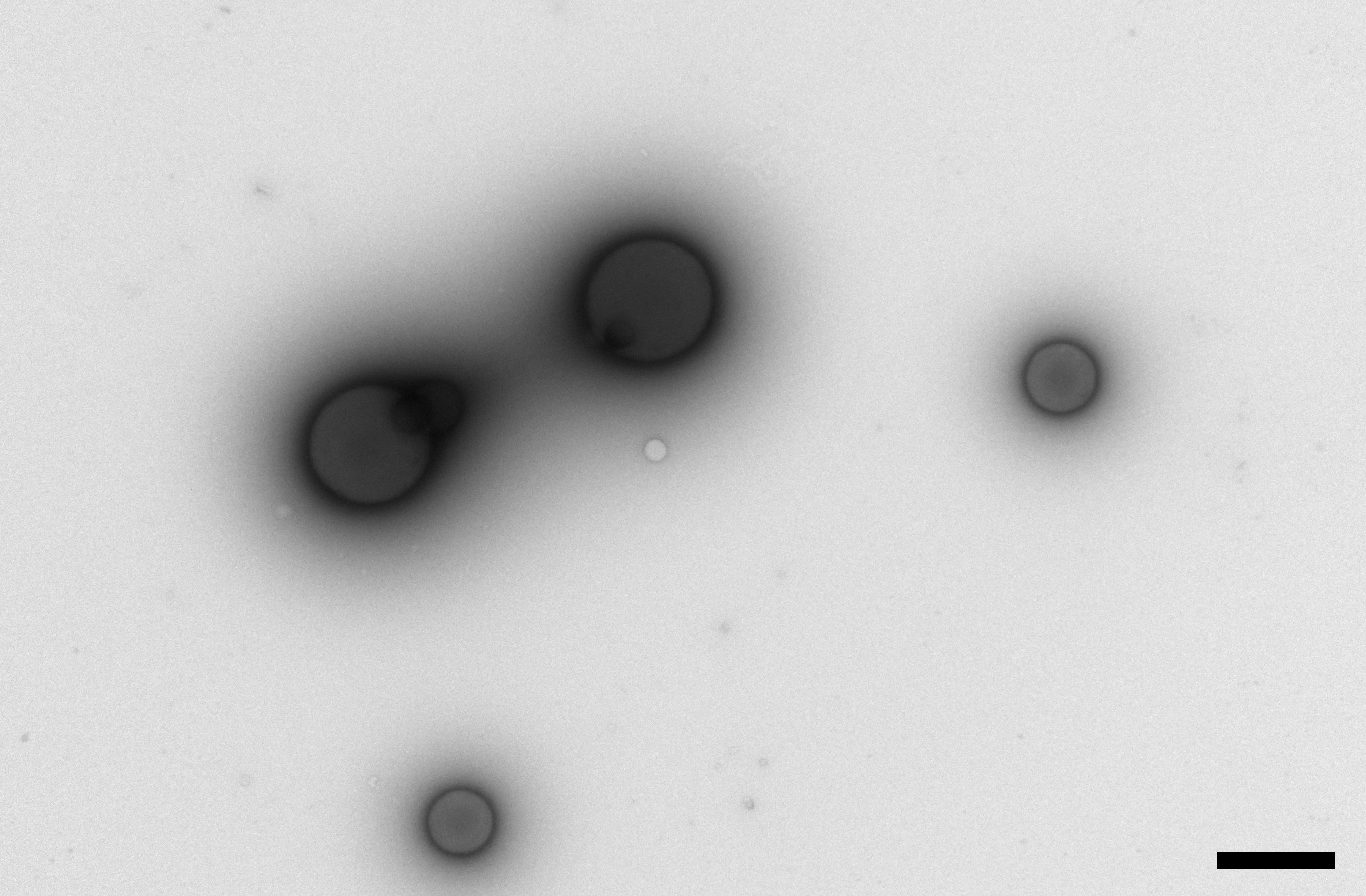

Supplement: SM-021-D5SM00213C-s001 [file SM-021-D5SM00213C-s001.zip › lpei np 1-5_010 copy-500nmscalebar.jpg]

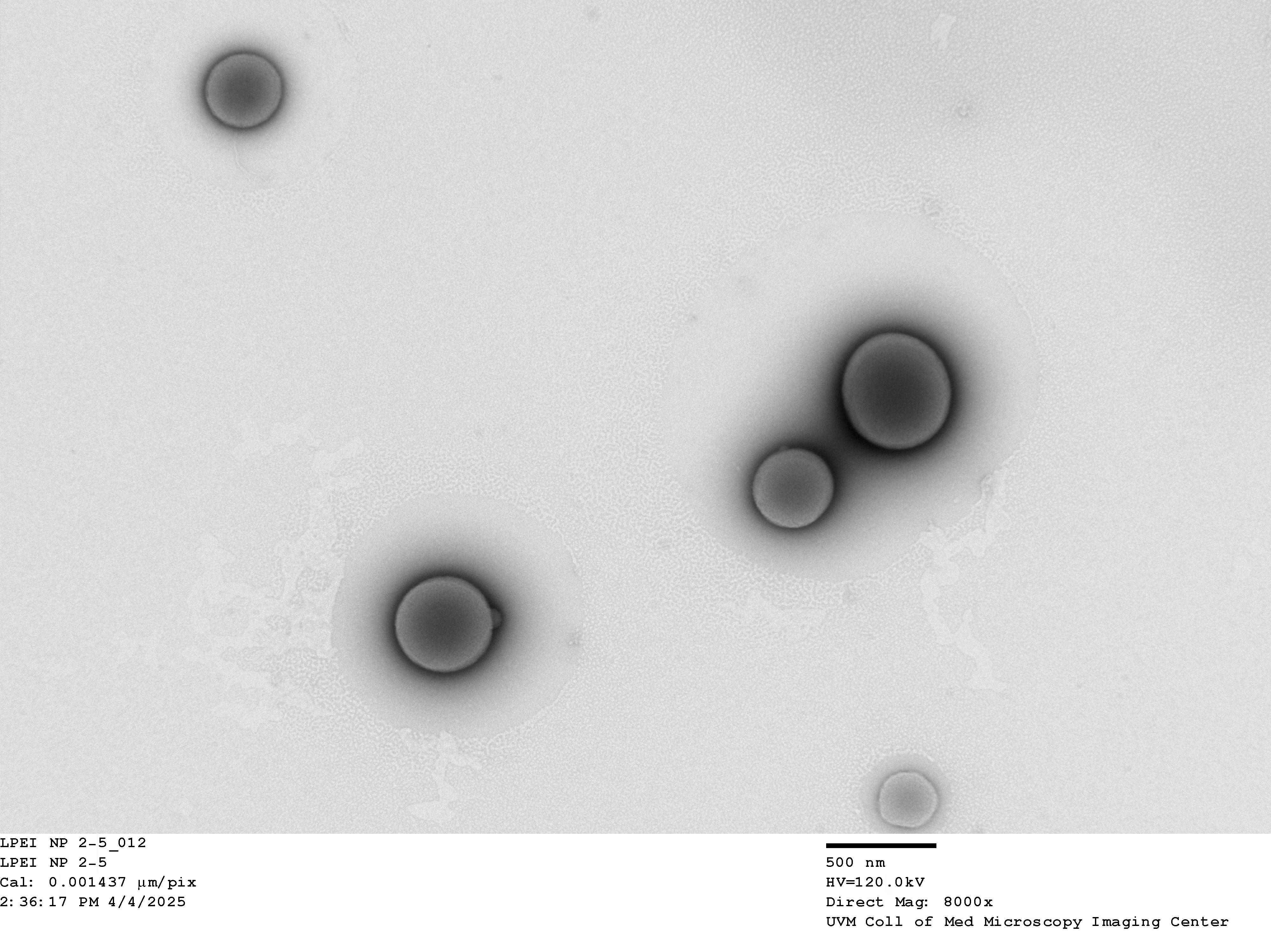

Supplement: SM-021-D5SM00213C-s001 [file SM-021-D5SM00213C-s001.zip › lpei np 2-5_012 copy.tif]

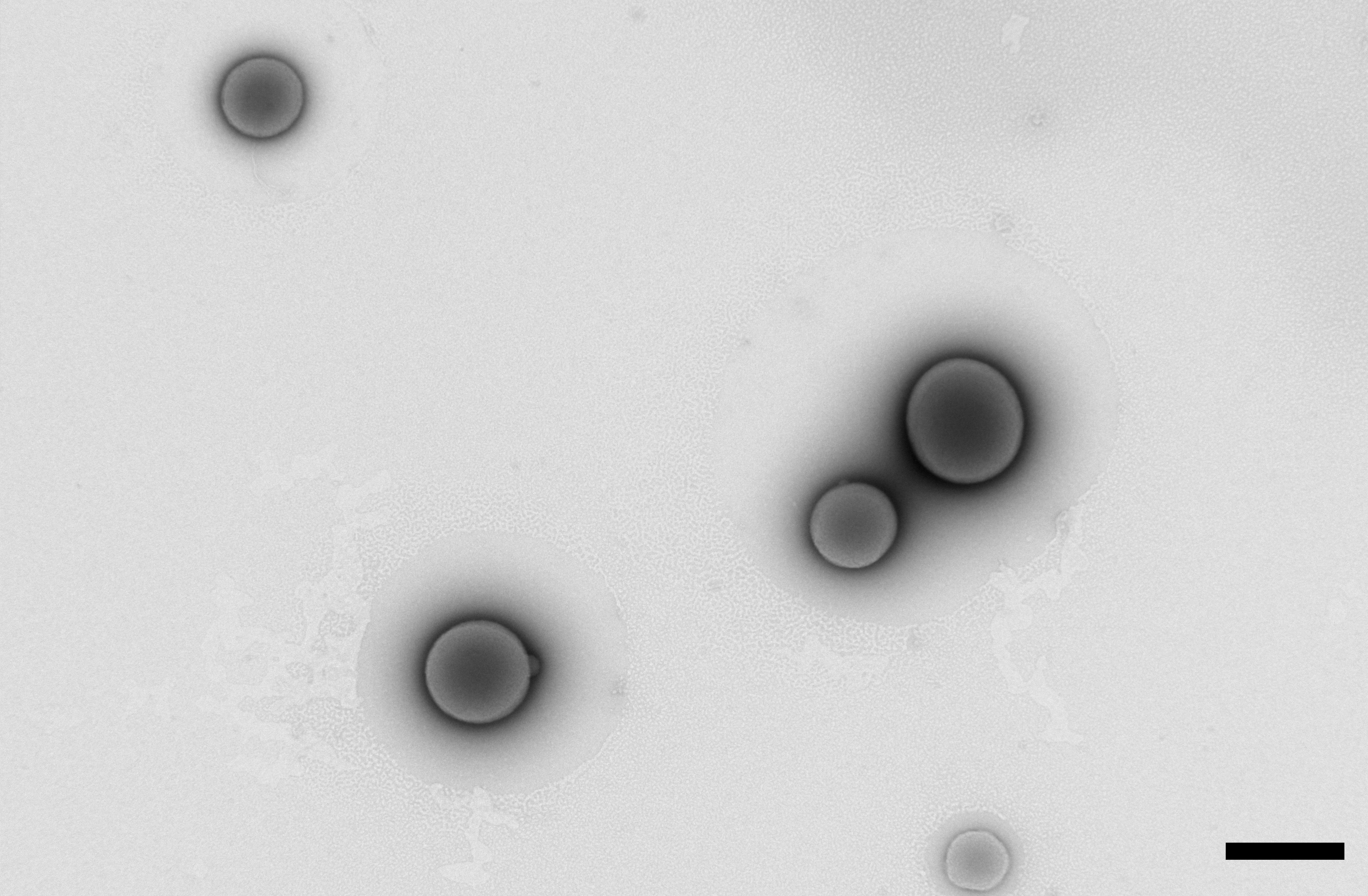

Supplement: SM-021-D5SM00213C-s001 [file SM-021-D5SM00213C-s001.zip › lpei np 2-5_012 copy-500nmscalebar.jpg]

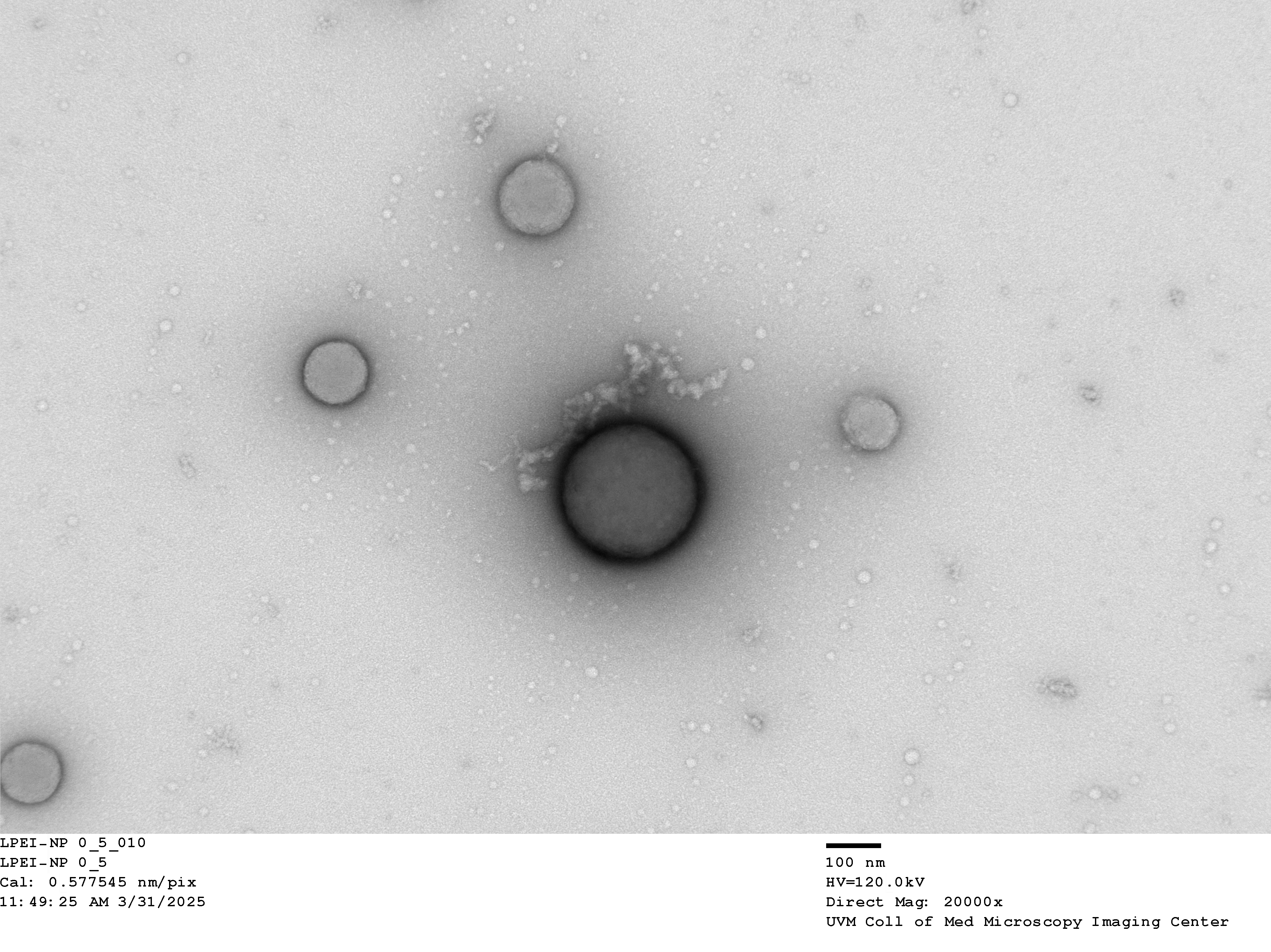

Supplement: SM-021-D5SM00213C-s001 [file SM-021-D5SM00213C-s001.zip › lpei-np 0_5_010 copy.tif]

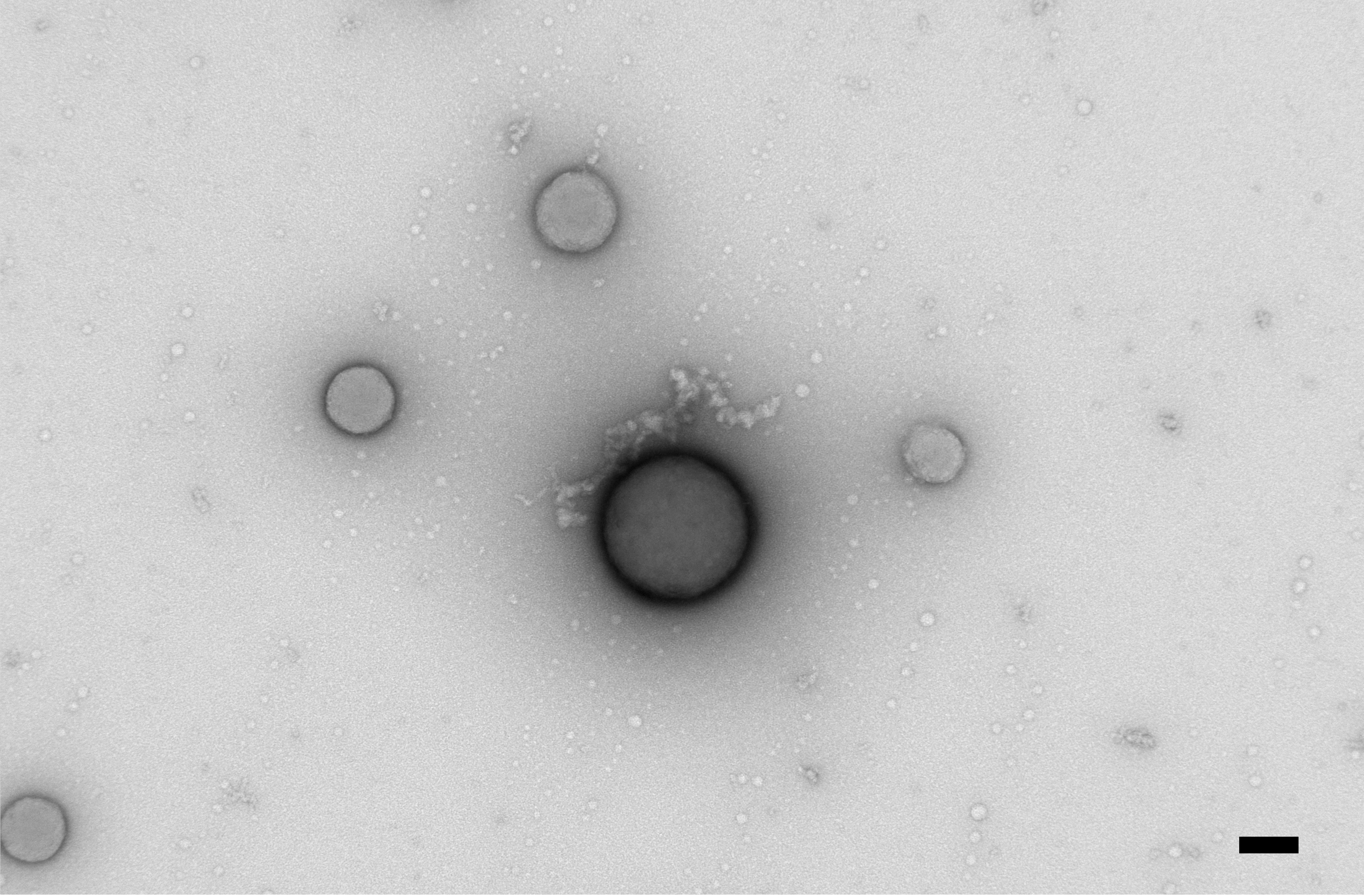

Supplement: SM-021-D5SM00213C-s001 [file SM-021-D5SM00213C-s001.zip › lpei-np 0_5_010 copy-100nmscalebar.jpg]

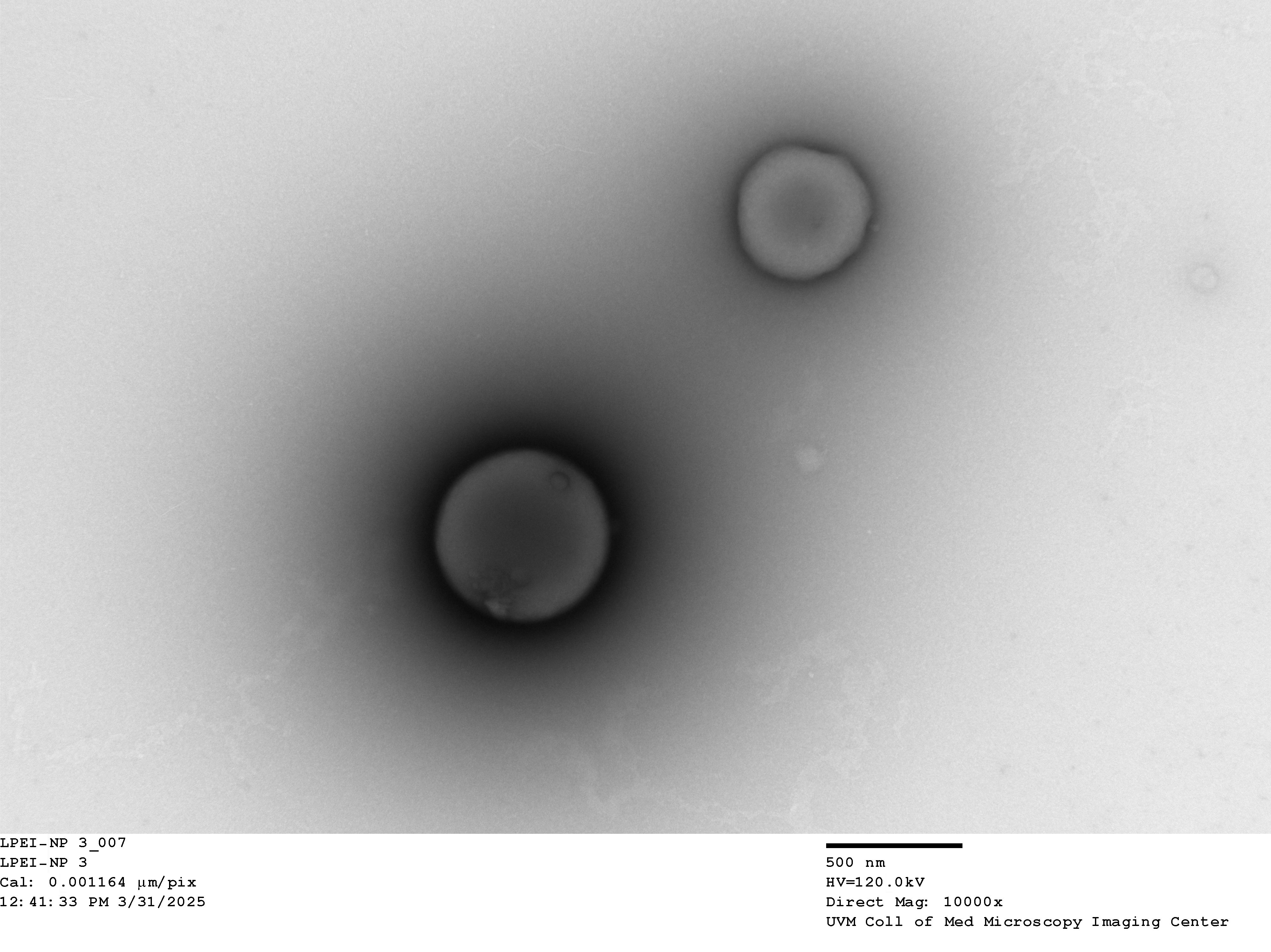

Supplement: SM-021-D5SM00213C-s001 [file SM-021-D5SM00213C-s001.zip › lpei-np 3_007 copy.tif]

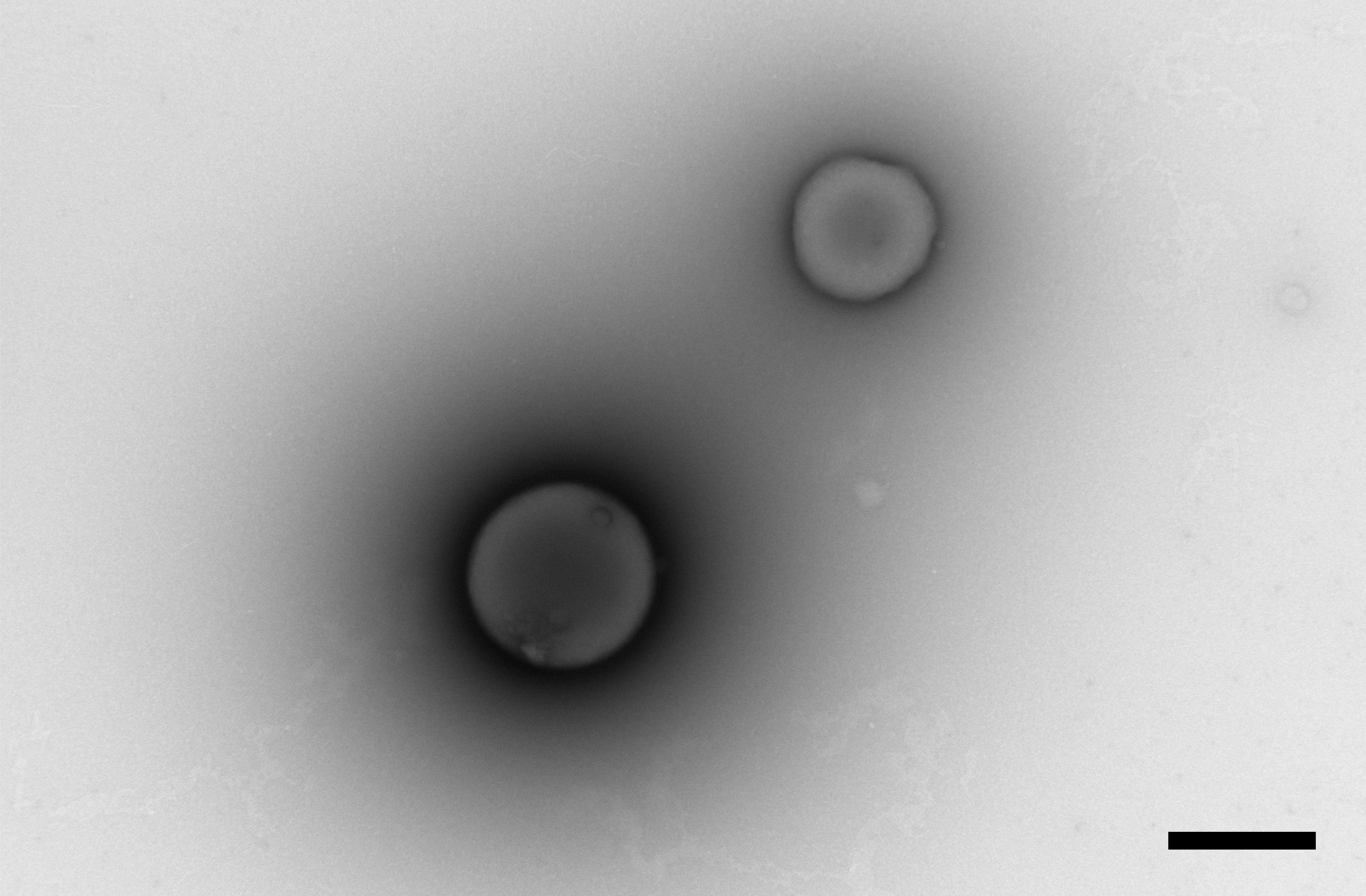

Supplement: SM-021-D5SM00213C-s001 [file SM-021-D5SM00213C-s001.zip › lpei-np 3_007 copy-500nmscalebar.jpg]

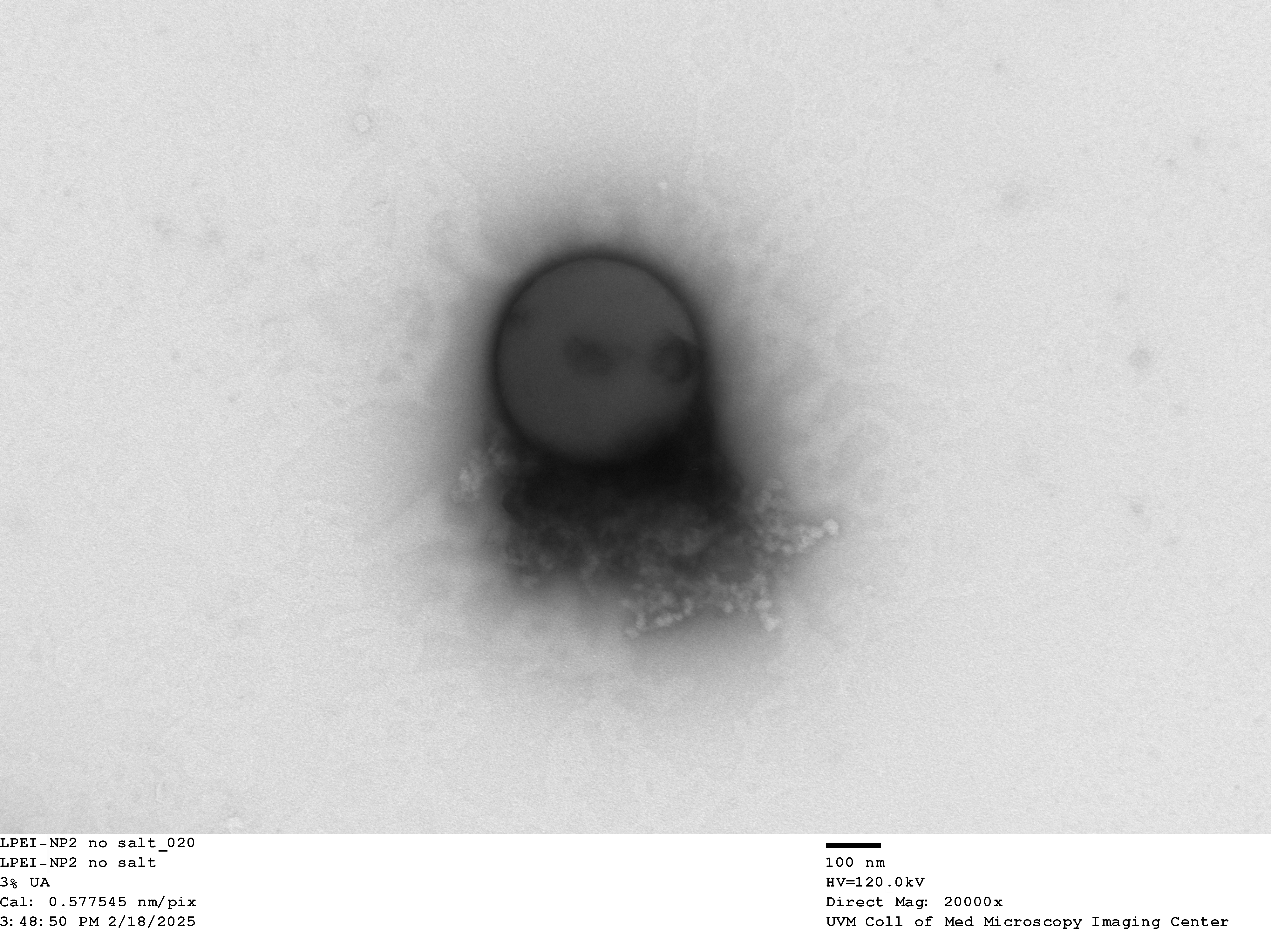

Supplement: SM-021-D5SM00213C-s001 [file SM-021-D5SM00213C-s001.zip › lpei-np2 no salt_020 copy.tif]

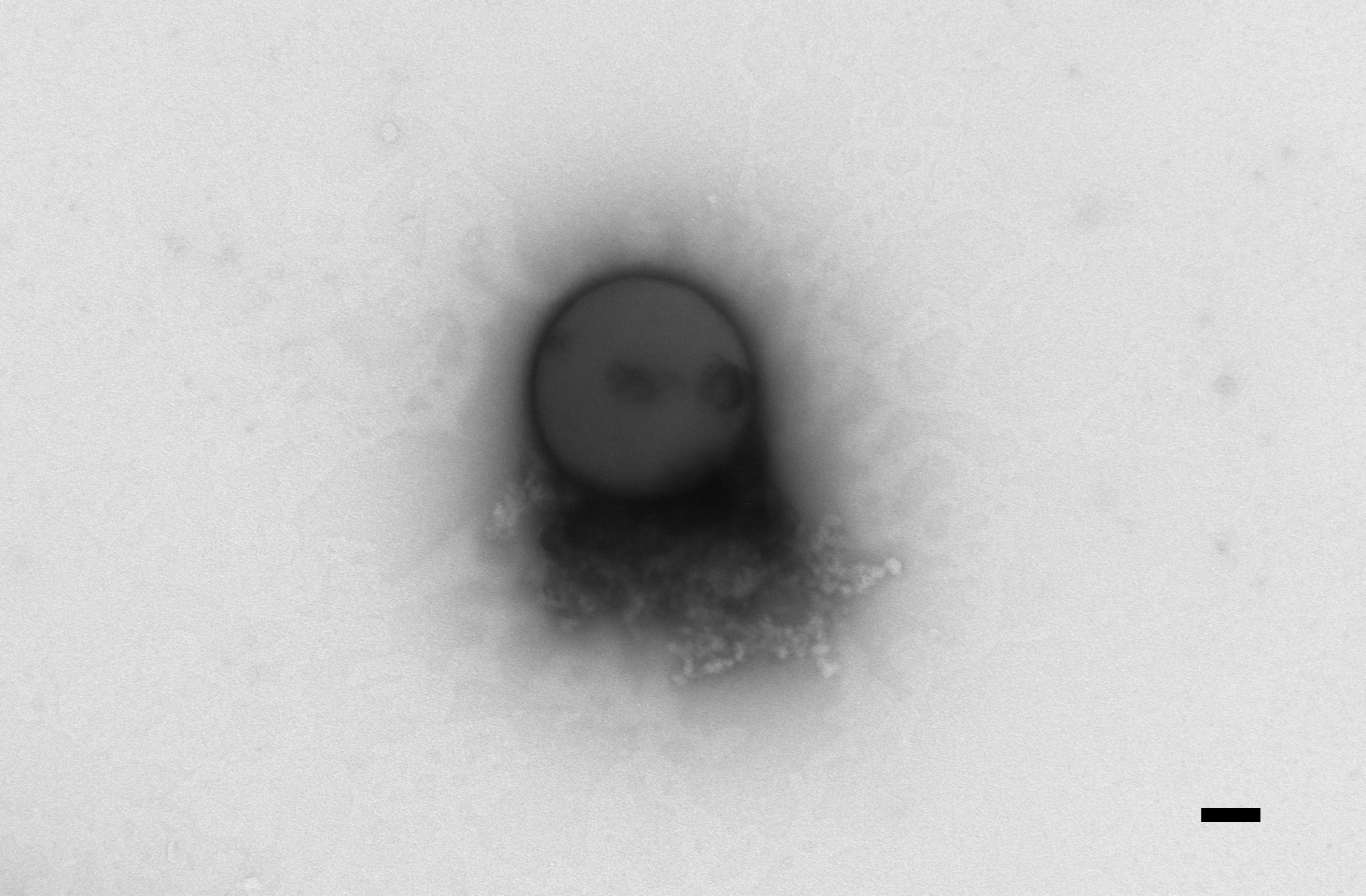

Supplement: SM-021-D5SM00213C-s001 [file SM-021-D5SM00213C-s001.zip › lpei-np2 no salt_020 copy-100nm-scalebar-crop.jpg]
