# Supplementary material for: Revealing two distinct molecular binding modes in polyethyleneimine–DNA polyplexes using infrared spectroscopy
Source: Soft Matter. 2025 Apr 26;21(21):4192–200. doi: 10.1039/d5sm00213c (PMC12053835; doi:10.1039/d5sm00213c)
Supplement: SM-021-D5SM00213C-s002 [file SM-021-D5SM00213C-s002.pdf]

Supplementary Information (SI) for Soft Matter.
This journal is © The Royal Society of Chemistry 2025

Revealing Two Distinct Molecular Binding Modes in Polyethyleneimine-DNA Polyplexes using Infrared Spectroscopy

Supporting Information

Rusul Mustafa†, Danielle Diorio†, Madeline Harper†,
and David Punihaole*,†

†Department of Chemistry, University of Vermont, Burlington, VT 05405
*Email: David.Punihaole@uvm.edu

Calculation method of N/P ratios. N/P ratio is defined as the number of amines of PEI to the number of phosphate of DNA. In this work, we calculated this ratio based on the final number of moles for both PEI and DNA while taking the number of amines and phosphate into consideration following these equations:

$$\text{Moles of PEI} = C_{PEI} \times Vol_t \times N_n \tag{S1}$$

$$\text{Moles of DNA} = C_{DNA} \times Vol_t \times N_b \tag{S2}$$

where C_{PEI} and C_{DNA} are the molar concentrations of PEI and DNA, Vol_t is the total volume of the sample, and N_n and N_b represent the number of monomer units in PEI and base pairs in DNA, respectively.

We varied the moles of PEI while keeping the moles of DNA fixed. We then calculated the N/P ratios following this equation:

$$\frac{N}{P} = \frac{\text{Moles of PEI}}{\text{Moles of DNA}} \tag{S3}$$

We followed this method for both UV-VIS, FTIR, and TEM measurements while adjusting the final concentrations of PEI and DNA accordingly.

Multivariate Curve Resolution Alternating Least Squares (MCR-ALS) Analysis. We utilized the MCR-ALS software built by Felten et al.[1] to conduct the MCR analysis on the FTIR spectra without the presence of NaCl. The software was able to extract two components, bound and unbound DNA, from a matrix that contains FTIR spectra of LPEI and BPEI polyplexes.

Sample preparation for transfection grade PEI. Transfection-grade PEI (MW 40,000 Da) was purchased from Kyforabio. A 1 mM polymer stock solution was prepared using 0.01 M phosphate buffer at pH 7.4. Upon adding PEI, the pH dropped to approximately 5.0. This stock was then diluted to prepare two polymer solutions at with varying concentrations. Each solution was then added dropwise to 1 mM DNA stock to achieve N/P ratios of 0.5 and 3. The samples were allowed to equilibrate for one hour before collecting IR measurements.

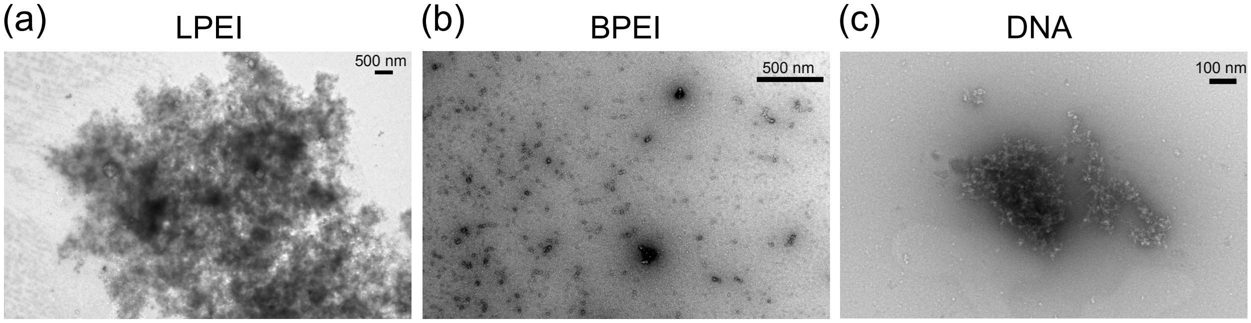

Figure S1: Representative TEM micrographs of (a) LPEI, (b) BPEI, and (c) DNA. The scale bar is 500 nm for (a) and (b). The scale bar is 100 nm for (c).

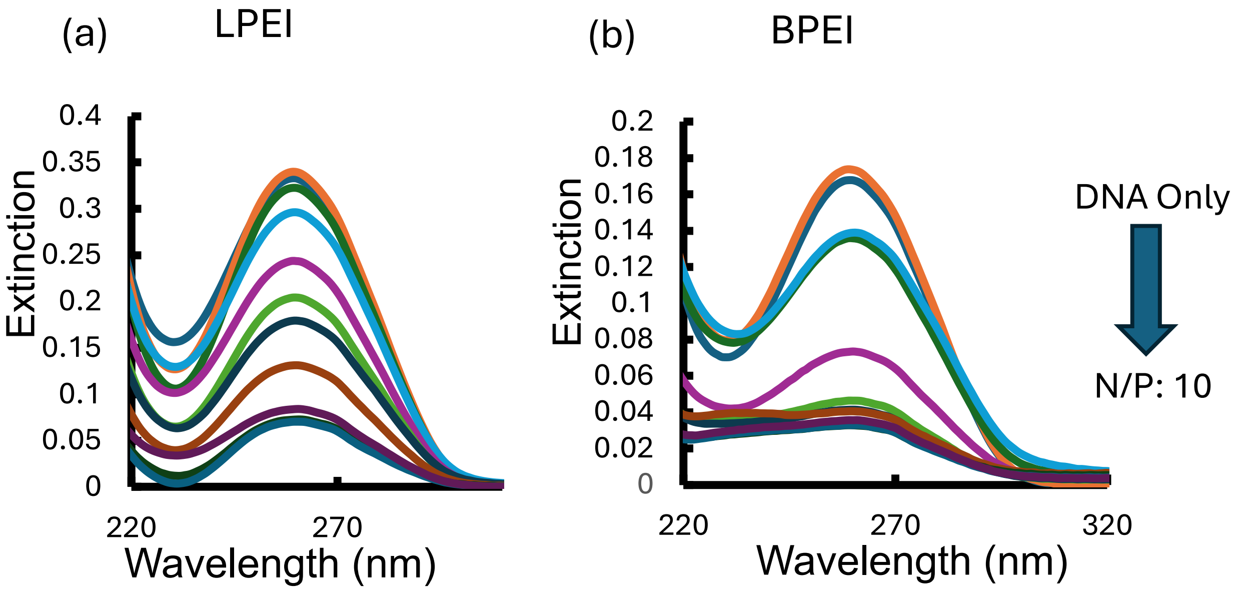

Figure S2: UV-Vis spectra of (a) LPEI and (b) BPEI polyplexes at N/P ratio of 0, 0.1, 0.5, 1, 1.5, 2, 2.5, 3, 5, 7, 10. The spectra were blank subtracted and baselined.

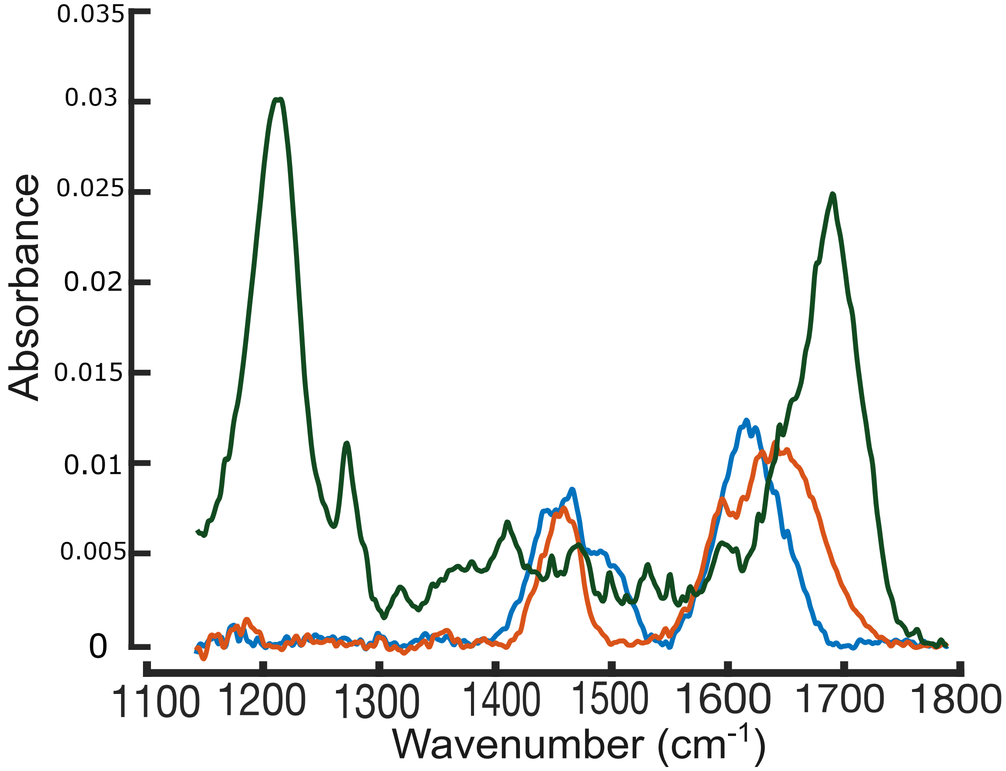

Figure S3: FTIR spectra of 2.0 mM LPEI (blue), 2.5 mM, BPEI (red), and 1.0 mM DNA (green) solutions in 0.01 M phosphate buffer, pH = 7.4. The spectral region of 1600 – 1700 cm^{-1} for LPEI and BPEI contains residuals of water bands. All spectra were blank subtracted and baselined.

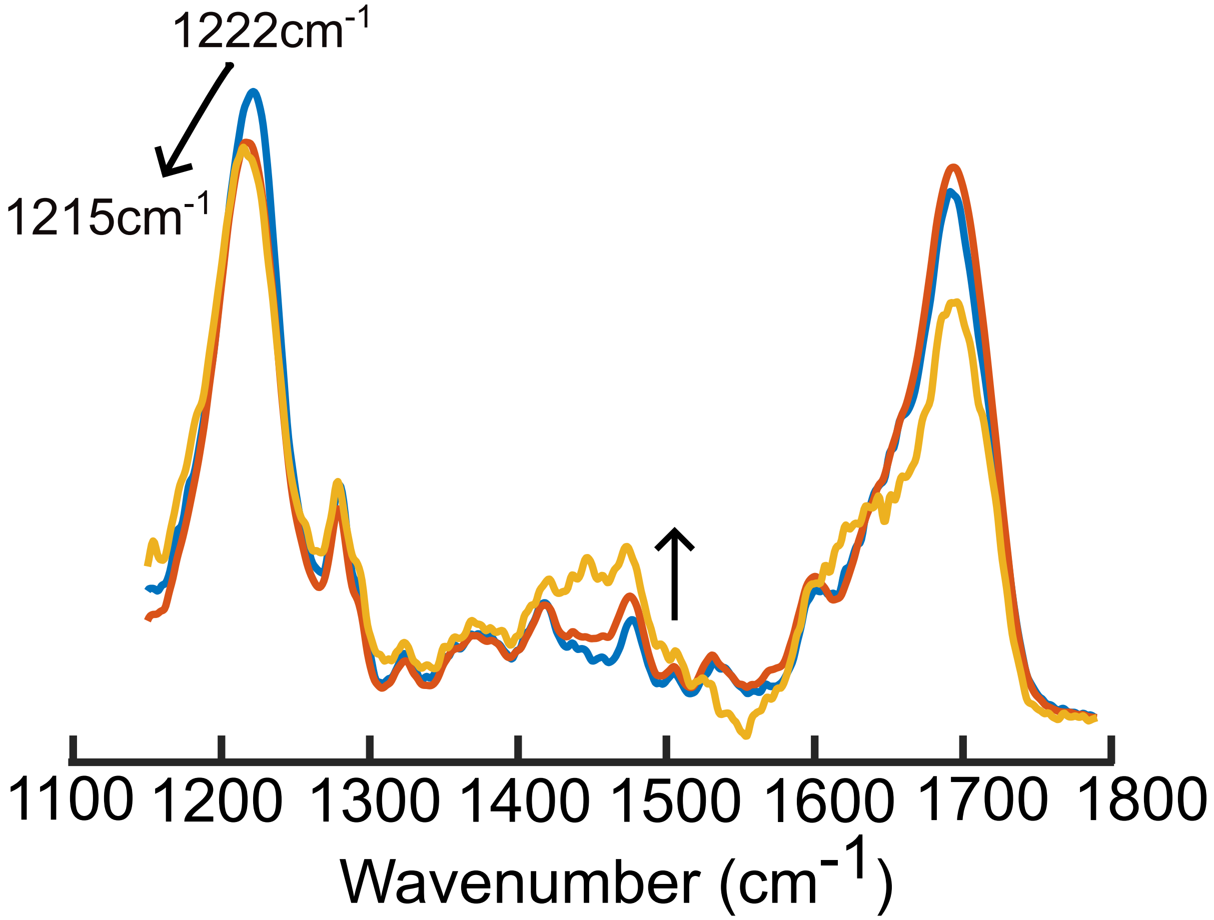

Figure S4: FTIR spectra of DNA control (blue) and transfection grade LPEI polyplexes with N/P of 0.5 (red) and 3 (yellow). All spectra were blank subtracted, baselined, and normalized to the total integrated area.

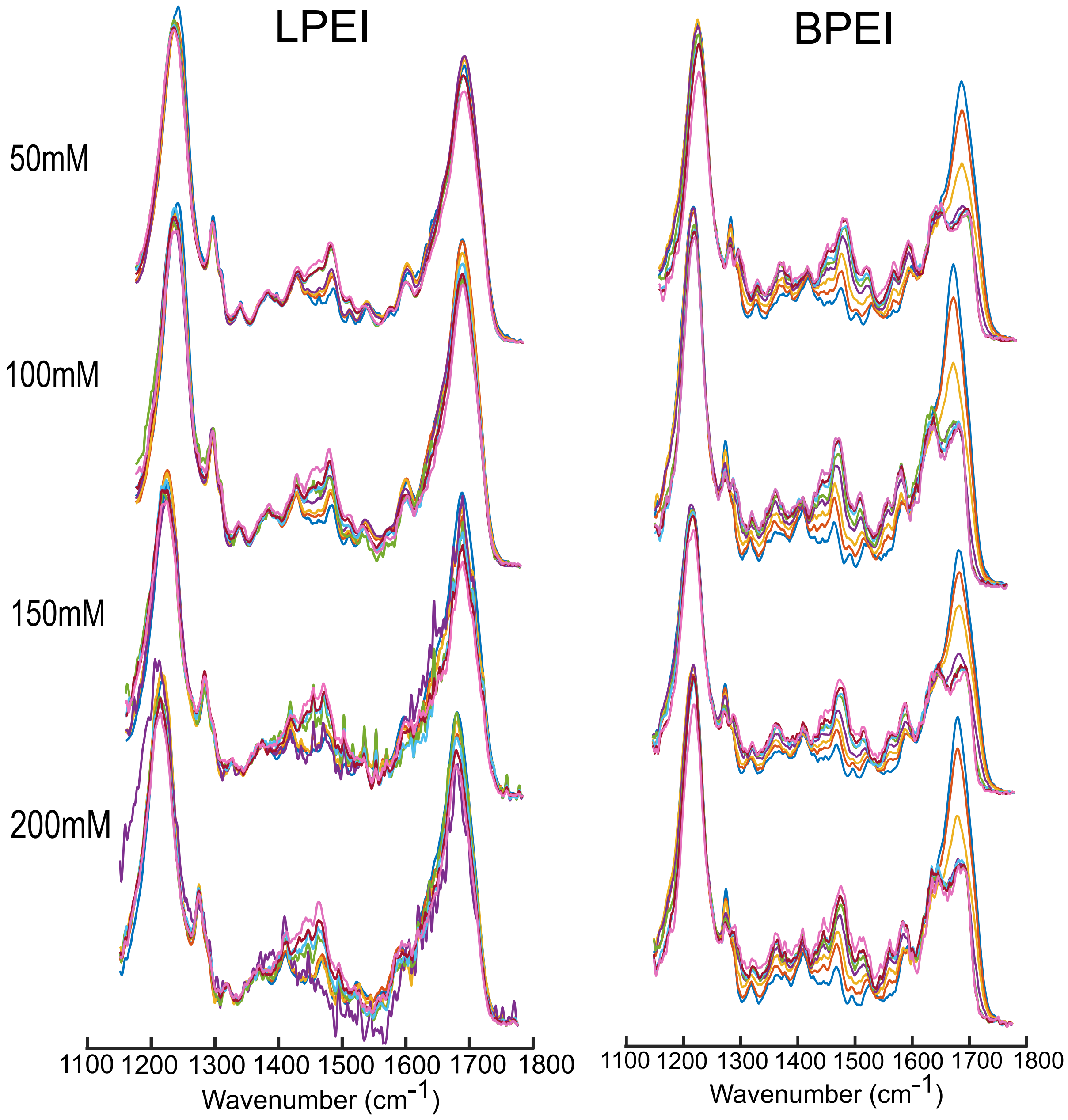

Figure S5: FTIR spectra of LPEI and BPEI polyplexes in the presence of varying NaCl concentrations. All spectra were blank subtracted, baselined, and normalized to the total integrated area.

Polymer	N/P Ratio	50 mM NaCl	150 mM NaCl	250 mM NaCl
LPEI	0.5	2.5%	0.4%	5.6%
	1.5	0.7%	27.1%	3.8%
	3	8.2%	0.4%	3.0%
	Average	5.7%		
BPEI	0.5	18.6%	6.1%	4.1%
	1.5	4.5%	5.8%	0.6%
	3	4.3%	2.3%	18.2%
	Average	7.2%		

Table S1: Percent error difference between two experimental replicates in the FTIR-based MCR-ALS analysis of LPEI and BPEI polyplexes at different NaCl concentrations. To calculate these errors, FTIR spectra of LPEI and BPEI polyplexes at N/P ratio of 0.5, 1.5, 2.5, and 3 in 50, 150, and 250 mM NaCl were measured. The spectra were then modeled using MCR-ALS analysis. The fraction bound value from the second replicate was subtracted from the first replicate, and the percent difference was averaged across all N/P ratios and NaCl concentrations to calculate the overall % error.

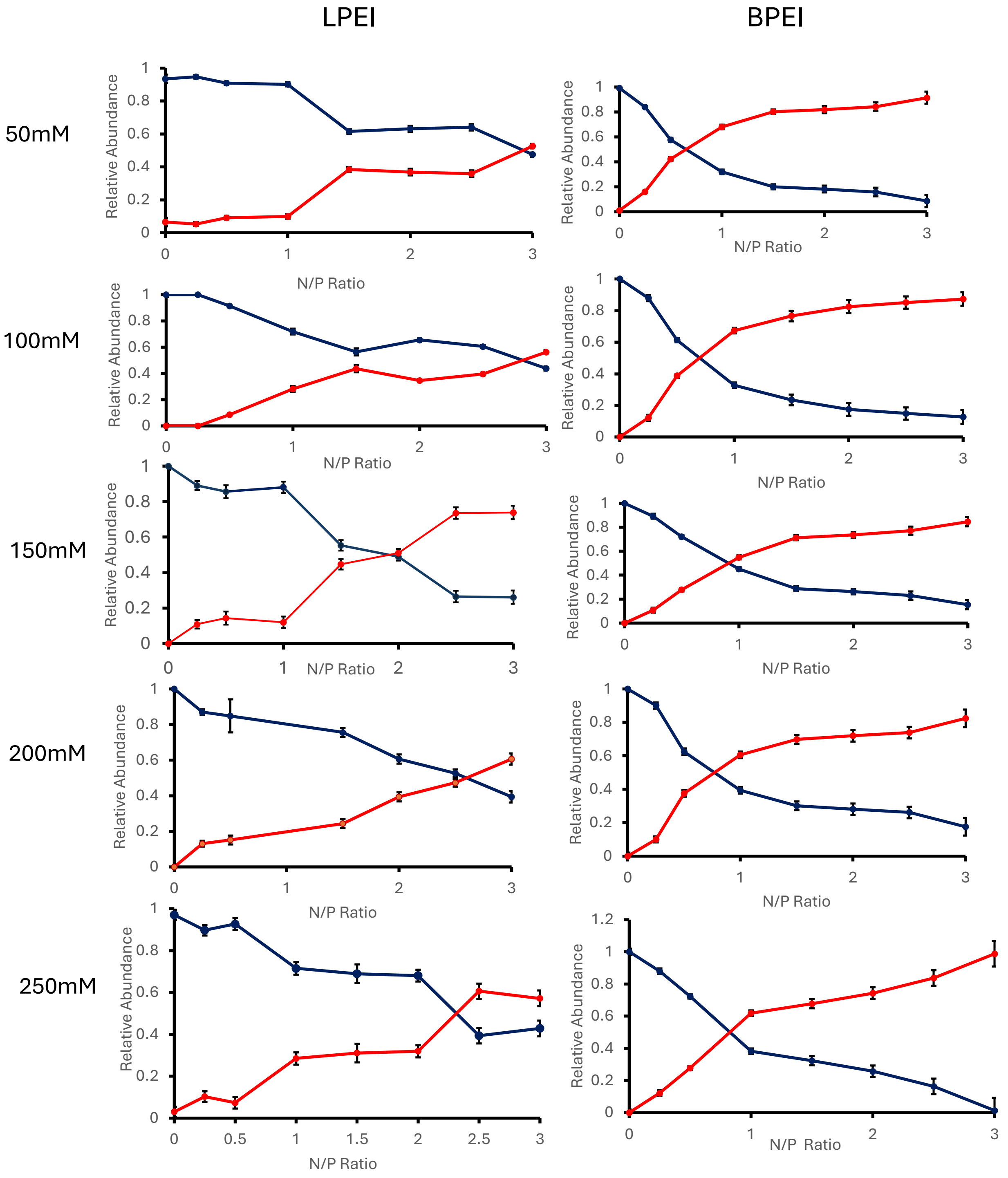

Figure S6: Concentration profiles of LPEI and BPEI complexes in varying concentrations of NaCl. The profiles represent the DNA bound (red) and unbound (blue) concentrations. The error bars represent the standard errors of the fit from the MCR-ALS analysis.

References

[1] Felten, J.; Hall, H.; Jaumot, J.; Tauler, R.; de Juan, A.; Gorzs, A. *Nat. Protoc.* **2015**, *10*, 217–240.
